# Study on the mechanism of 18β-glycyrrhetinic acid inhibiting the proliferation of renal cancer cells by inducing autophagy through the miR-27a-5p/LC3 axis

**DOI:** 10.3389/fonc.2026.1762770

**Published:** 2026-02-27

**Authors:** Shumin Jia, Lei Zhang, Yahong Li, Duojie Xu, Yi Yang, Ziying Zhou, Wenjing Liu, Jianan Zhao, Ling Yuan, Yi Nan

**Affiliations:** 1 Key Laboratory of Dryness Syndrome in Chinese Medicine, Ministry of Education, Ningxia Medical University, Yinchuan, China; 2 Traditional Chinese Medicine College, Ningxia Medical University, Yinchuan, China; 3 College of Pharmacy, Ningxia Medical University, Yinchuan, China

**Keywords:** 18β-glycyrrhetinic acid, autophagy, miR-27a-5p/LC3 axis, proliferation, renal carcinoma

## Abstract

**Background:**

Renal carcinoma is a common, aggressive urinary tract malignancy with notable clinical challenges such as severe treatment toxicity and poor patient outcomes; 18β-glycyrrhetinic acid (18β-GA), an active component of Chinese herb Glycyrrhiza uralensis, has potent anti-tumor activity, while its role and molecular mechanisms in renal cancer remain elusive.

**Aim:**

This research investigates the mechanism through which 18β-GA suppresses renal cancer cell proliferation.

**Methods:**

Combining whole transcriptome sequencing and network pharmacology, we identified 18β-GA-regulated key molecule miR-27a-5p and its core renal cancer targets; Cell assays confirmed 18β-GA-mediated suppression of renal cancer cell proliferation. Lentivirus-mediated miR-27a-5p modulation verified its role in renal cancer proliferation, and Western blot detection of autophagy marker LC3 expression clarified the miR-27a-5p/LC3 axis involvement in the anti-renal cancer effects of 18β-GA.

**Results:**

Research shows 18β-GA may exert anti-renal cancer effects by targeting HMOX1, HCK, CASP1 and IDO1, with its mechanism linked to the autophagy pathway via functional enrichment analysis; whole transcriptome sequencing identified miR-27a-5p as the most significantly altered by 18β-GA in renal cancer cells. Experimental verification confirmed that 18β-GA downregulates miR-27a-5p to elevate the autophagy marker LC3II/LC3I ratio, activate autophagy, reduce 786-O and ACHN cell viability, promote apoptosis, inhibit colony formation, and thus suppress renal cancer cell proliferation.

**Conclusion:**

18β-GA induces autophagy and inhibits proliferation of renal cancer cells by down-regulating miR-27a-5p and relieving its inhibition on the LC3-mediated autophagy pathway, suggesting that the miR-27a-5p/LC3 axis may be a key target for 18β-GA in the treatment of renal cancer.

## 1 Introduction

Renal cancer is one of the common urinary system cancers worldwide (1, 2). In 2020, renal cancer accounted for about 431,000 new cases globally, making it the 14th most common malignant tumor by incidence. There were approximately 179,000 deaths, ranking 15th in terms of mortality rate (3). Renal cancer has clear risk factors, including smoking, obesity and hypertension (4). Diagnosing early-stage renal cancer is challenging due to a lack of symptoms, as typical signs of renal cell carcinoma—abdominal pain, hematuria, and a palpable abdominal mass—usually emerge only in advanced stages (5). Currently, the treatment options for renal cancer mainly include radical nephrectomy (6), partial nephrectomy (7), chemotherapy (8), immune checkpoint therapy (9) etc. Despite nephrectomy being the gold standard for renal cancer treatment, some patients still face recurrence, and chemotherapy’s side effects significantly impact their quality of life (10). Hence, there is a pressing demand for the discovery of natural agents that are effective against renal cancer with minimal toxicity.

Traditional Chinese Medicine (TCM) is now widely used for complex diseases like cancer, and a kidney-tonifying and spleen-strengthening formula can inhibit clear cell renal cell carcinoma proliferation by regulating the immune rejection state of the tumor microenvironment (11). The extract of Sinomenium acutum, sinomenine, can enhance autophagy and promote apoptosis of RCC cells (12). Curcumin inhibits the viability of ACHN cells by suppressing the AKT/mTOR pathway, inducing apoptosis and autophagy (13). Traditional Chinese medicine extracts such as tetrandrine (14), salidroside (15), tetramethypyrazine (16), Poria acid (17), dendrobine (18) and paeonol (19) can exert anti-cancer effects on RCC. Furthermore, these TCM extracts exhibit significant anti-tumor effects in cancer treatment and can reduce liver and kidney damage. Thus, investigating the anti-cancer properties of TCM holds substantial potential for clinical translation (17).

18β-glycyrrhetinic acid (18β-GA), a key bioactive compound in licorice, exhibits various pharmacological properties such as anti-inflammatory, hepatoprotective, and anti-tumor effects (20–24). Recent studies have shown that 18β-GA has notable antitumor effects on several human cancers, such as gastric (25), lung (22), breast (26), liver (27), and ovarian (28) cancers. Our team has previously demonstrated that 18β-GA enhances autophagy and suppresses gastric cancer cell proliferation by modulating the miR-328-3p/STAT3 (29) and miR-345-5p/TGM2 signaling pathways (25). In addition, 18β-GA can also regulate the mitochondrial ribosomal protein L35-related apoptosis signaling pathway to induce apoptosis of gastric cancer cells and thereby inhibit their proliferation (30). Moreover, GA can accelerate the excretion of toxins by regulating P-glycoprotein, and has the characteristics of good pharmacological activity and few adverse reactions (31). Research shows 18β-GA exerts anti-tumor effects on multiple cancers possibly via autophagy; its exact anti-renal cancer mechanism is unclear, but it is hypothesized to be a potential agent for renal cancer prevention and treatment.

Renal cancer is characterized by unique metabolic changes during its onset and development, including enhanced aerobic glycolysis, pentose phosphate pathway activity, fatty acid biosynthesis, and glutamine and glutathione metabolism. Simultaneously, the tricarboxylic acid cycle, fatty acid β-oxidation, and oxidative phosphorylation are suppressed. These coordinated alterations in cellular metabolism are commonly termed “metabolic reprogramming” (32). Autophagy is a regulated process essential for cellular homeostasis, removing damaged organelles and proteins (33). Autophagy is categorized into three types based on the transport mechanism of the degradable material: chaperone-mediated autophagy, microautophagy, and macroautophagy (34). This paper focuses on macroautophagy, the most prevalent form. Research indicates that autophagy suppresses renal cancer cell proliferation (35), with compounds like gallic acid, silybin, and capsaicin inducing autophagy via various pathways to inhibit the progression, migration, invasion, and metastasis of these cells *in vivo* (36–38). In addition, microRNA-100 enhances autophagy in renal cancer cells and inhibits their migration and invasion (39). Given the metabolic traits of renal cancer cells, we hypothesize autophagy is critical for regulating their growth and metabolic homeostasis, and that 18β-GA may inhibit renal cancer progression by modulating autophagy.

MicroRNAs (miRNAs) are evolutionarily conserved, non-coding RNA molecules that play critical roles in the development and advancement of cancer (40). miRNAs influence protein expression and regulate essential cellular functions and signaling pathways by binding to target mRNAs (41). Accumulating evidence shows miRNAs participate in renal cell carcinoma pathogenesis; miR-203, miR-384 and miR-186 inhibit tumor progression by targeting specific genes, with miR-203 suppressing renal cancer cell proliferation, migration and invasion via FGF2 targeting (42); miR-384 reduces tumor growth and invasiveness by downregulating AEG-1 (43); and miR-186 hinders cancer cell proliferation and metastasis through SENP1 modulation (44). This study utilized whole-transcriptome sequencing to examine miRNA expression in renal cancer cells after 18β-GA treatment, identifying miR-27a-5p as the most differentially expressed miRNA. Therefore, this study concentrated on miR-27a-5p to clarify its biological function and molecular mechanisms in the progression of renal cell carcinoma.

**Figure 1:**
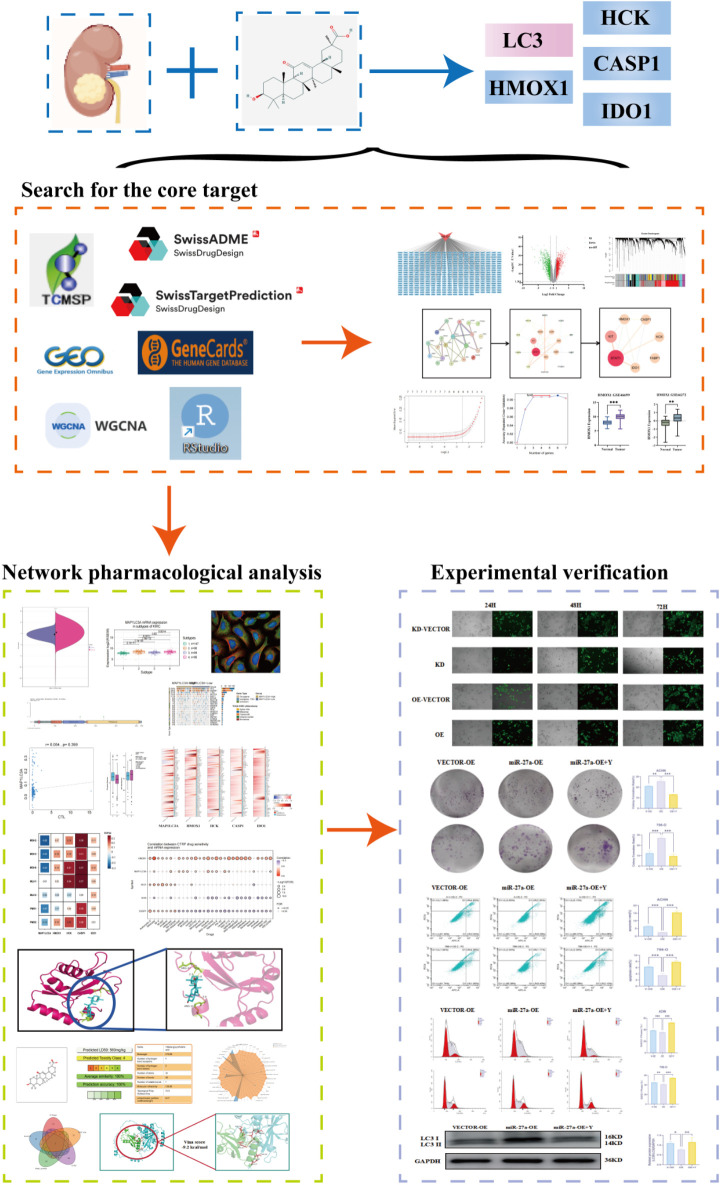
The flow chart.

This research integrates network pharmacology with *in vitro* cell experiments to investigate the mechanism by which 18β-GA suppresses renal cancer proliferation. Network pharmacology aligns with TCM’s holistic systems perspective by elucidating disease-syndrome-prescription interactions via biological networks, providing a novel approach for investigating TCM mechanisms and advancing new drug development (45). We propose that 18β-GA may suppress renal cancer cell proliferation by modulating the miR-27a-5p/LC3 signaling pathway and influencing autophagy, offering a theoretical foundation for its use in renal cancer treatment. The research approach is illustrated in **Figure 1**.

## 2 Materials and methods

### 2.1 18β-GA targets and renal cancer targets acquisition

First, the targets of 18β-GA were predicted in the Swisstargetprediction database and the PharmMapper database, and the drug-target network diagram was drawn using Cytoscape software. Then, the targets of renal cancer were predicted through the GeneCards database. In the GEO database, the keyword “renal cancer” was used for retrieval, and the dataset GSE46699 was selected. The conditions P < 0.05 and |LogFC|≥1 were set for differentially expressed genes, and a volcano plot was drawn using Graphpad. At the same time, the intersection of the results of the three was taken to obtain the intersection target diagram of 18β-GA-renal cancer-GEO.

### 2.2 WGCNA analysis

The GSE46699 dataset was subjected to differential gene analysis through the oebiotech website. Subsequently, the intersection of drug genes, renal cancer genes, GEO differential genes, and WGCNA genes was taken. Then, the corresponding GEO data of the intersection targets were imported into WeishengXin to generate a heatmap. The intersection targets were sorted by LogFC values and a bar chart was drawn in WeishengXin. Meanwhile, principal component analysis was conducted in the PCoA of the Bioladder website.

### 2.3 Enrichment analysis

The targets identified in the prior step were uploaded into the String database, followed by data retrieval and subsequent import into Cytoscape for protein-protein interaction network analysis. Genes with a Degree ≥ 4 were selected as core targets, and the Degree values were input into the Chiplot website for visualization. The GEPIA 2.0 website was used to obtain the correlations among the core targets, and then the intra-group correlation heat map was drawn using Chiplot. The overlapping target genes were uploaded to the DAVID database to perform Gene Ontology (GO) enrichment analysis and Kyoto Encyclopedia of Genes and Genomes (KEGG) pathway analysis. The top-ranked results of the GO analysis were imported into the Sangerbox website to draw bar charts, and the KEGG results were used to draw enrichment analysis circle diagrams. After sorting by P-value, the Sankey diagram of the pathways was drawn using Microbiomics.

### 2.4 Analysis of machine learning algorithms

We conducted Lasso, SVM and Random Forest analyses on the intersectional targets obtained from protein-protein interactions using R language to explore more clinically significant targets. Meanwhile, we constructed training and validation sets in Graphpad to verify the analysis results.

### 2.5 Analysis of the clinical significance of core target genes

The clinical relevance of the core targets was analyzed as follows. Firstly, the Sagnerbox online analysis tool was used to obtain the mRNA expression of the core targets. Then, the targets were input into the GEPIA2.0 website to draw the copy number graph. Next, on the UALCAN website, the tumor type was selected as “Kidney renal clear cell carcinoma” to obtain the box plot of the target protein expression. Subsequently, on the GSCA online website, “Expression” and “Expression & Subtype” were selected to analyze the expression of the targets in different subtypes of renal cancer. Finally, the genes were input into the GEPIA2.0, and the stage graph of renal cancer was drawn in the Expression Analysis.

### 2.6 Immunohistochemical and survival prognosis analysis of core targets

The core targets were entered into the Human Protein Atlas (HPA) database, where the TISSUE option was set to “KIDNEY” and the PATHOLOGY section was configured to “CANCER” with the subtype specified as “renal cancer,” enabling retrieval of immunohistochemical expression profiles in both normal kidney and renal cancer tissues. Immunofluorescence images of the target proteins were acquired using the SUBCELL module of the HPA database. Additionally, the targets were submitted to UALCAN, selecting the “Kidney renal clear cell carcinoma” dataset to evaluate their survival prognosis.

### 2.7 The relationship between core target mutations and renal cancer

The core targets were uploaded to the GSCA website, and the tumor type was specified as KIRC. In the “Mutation” module’s “SNV summary” section, the mutation sites and types of SNVs were obtained, and a heat map of the harmful SNV mutation frequency in renal cancer was also obtained. In the “CNV” module, bubble plots depicting heterozygous and homozygous copy number variations of the core targets were generated. Subsequently, the core genes were analyzed using the CAMOIP website to obtain the mutation correlation map and the box plot illustrating microsatellite instability (MSI) expression levels. The occurrence of tumors is related to gene mutations in the body, but the HHR and MMR systems in the body help to repair them. The core genes and the genes of the HHR and MMR repair systems were input into the GEPIA2 website, with “KIRC Tumor” selected. Only the R value data was taken, and the obtained data was imported into the Chiplot website for plotting. A heat map of core genes related to the HRR and MMR repair systems was obtained.

### 2.8 The relationship between core target methylation and renal cancer

The core target genes were introduced into the UALCAN platform utilizing TCGA dataset for further analysis. The TCGA dataset selected “Kidney renal clear cell carcinoma” and “Methylation” as the results to obtain the gene methylation expression level graph. The TIDE website can analyze the relationship between the methylation level of core targets and survival prognosis. On the TIDE platform, the “Query Gene” function was selected to upload the core targets, and the “CTL Cor” results were retrieved to analyze the association between levels of gene methylation and markers of cytotoxic T lymphocyte (CTL). The “Risk” result was the survival curves of high methylation and low methylation subgroups of genes.

### 2.9 Immune relevance of core targets

Access the Sangerbox website and select immune infiltration analysis under pan-cancer analysis. Choose TCGA-KIRC in ‘CODE’ to generate scatter plots for the stromal, immune, and estimated scores of the core targets. In the pan-cancer analysis, select immune checkpoint gene analysis and choose ‘KIRC’ in ‘CODE’ to generate the correlation heatmap between core targets and immune checkpoints. Access the TISCH website and choose the ‘KIRC_GSE121636’ dataset to retrieve core gene expression data in immune cells. Subsequently, examine the expression of immune-related cells and core genes in renal cancer. On the TIMER2.0 website, enter the core targets, select “Cancer associated fibroblast, Macrophage, Monocyte, T cell CD8+”, filter out the “KIRC” data and import it into Chiplot to draw the correlation heatmap to show the correlation.

### 2.10 The correlation between core targets and antitumor drug therapy

The core targets were uploaded to the TISIDB platform, and the ‘Drug’ option was selected to create the immunotherapy-related network map of the core genes. The CAMOIP website’s “Immune Infiltration” module was used to analyze the associations between core gene expression levels and key immune-related cytokines INF-γ and TGF-β. Lastly, the key genes were input into the “Drug” section of the GSCA database to obtain drug sensitivity data and matched mRNA expression levels from the GDSC and CTRP databases.

### 2.11 Molecular docking

Input the core targets respectively into the PDB database, and download the 3D structure files of the core targets. Use Open Babel software to convert the file format to PDB format. Download the SDF structure of 18β-GA from PubChem and analyze it on the CB-Dock2 website. Record the corresponding Vina score values and import the data into ChiPlot to draw a heatmap for display. At the same time, utilize PyMOL software to visualize the molecular docking outcomes.

### 2.12 Toxicity analysis of 18β-GA

Next, we conducted toxicity predictions for 18β-GA on the ProTox 3.0 - Prediction Of Toxicity Of Chemicals website and summarized the results.

### 2.13 Acquisition of upstream transcription factors and downstream target proteins

Using the TF-Target Finder database, we identified the upstream transcription factors of MAP1LC3B. Then, we searched for the downstream targets of MAP1LC3B through the GeneMANIA database, HitPredict database and STRING database, and determined the downstream targets by taking the intersection of the three databases. We analyzed the molecular docking ability of MAP1LC3B and its downstream target proteins through the GRAMM database, and then analyzed the binding energy through the PDBe database. Finally, we visualized the docking results using PyMOL software. The websites related to network pharmacology prediction are shown in **Table 1**.

**Table 1:** Relevant websites for network pharmacology prediction.

Database	Web address
Swisstargetprediction	http://www.swisstargetprediction.ch/
PharmMapper	https://lilab-ecust.cn/pharmmapper/submitfile.html
GeneCards	https://www.genecards.org/
GEO	https://www.ncbi.nlm.nih.gov/geo/
Oebiotech	https://cloud.oebiotech.com/
Weishengxin	https://www.bioinformatics.com.cn/
Bioladder	https://www.bioladder.cn/web/#/pro/cloud
String	https://cn.string-db.org/
Chiplot	https://www.chiplot.online/
GEPIA2.0	http://gepia2.cancer-pku.cn/#index
DAVID	https://davidbioinformatics.nih.gov/
Sangerbox	http://sangerbox.com/home.html
UALCAN	https://ualcan.path.uab.edu/index.html
GSCA	https://guolab.wchscu.cn/GSCA/#/
HPA	https://www.proteinatlas.org/
CAMOIP	http://www.zjyy-oncology.com:20002/
TIDE	http://tide.dfci.harvard.edu/
TIMER2.0	http://timer.cistrome.org/#tab-2767-2
TISCH	http://tisch.comp-genomics.org/
TISIDB	http://cis.hku.hk/TISIDB/simple_search_result.php
PDB	https://www.rcsb.org/
PubChem	https://pubchem.ncbi.nlm.nih.gov/
CB-Dock2	https://cadd.labshare.cn/cb-dock2/php/blinddock.php
ProTox 3.0 - Prediction Of Toxicity Of Chemicals	https://tox.charite.de/
TF-Target Finder	https://jingle.shinyapps.io/TF_Target_Finder/
GeneMANIA	https://genemania.org/
HitPredict	https://www.hitpredict.org/
GRAMM	https://gramm.compbio.ku.edu/
PDBe	https://www.ebi.ac.uk/pdbe/
ENCORI	https://rnasysu.com/encori/panCancer.php

### 2.14 Total transcriptome sequencing analysis

To identify differentially expressed miRNAs between renal cancer cells (control group) and 18β-GA-treated renal cancer cells (experimental group), comprehensive transcriptome sequencing was performed with three biological replicates per group (n=3), where all replicate samples were derived from independent cell culture batches. Sequencing results were visualized using volcano plots, bar graphs, and heatmaps to intuitively display differential miRNA expression profiles. Subsequently, functional enrichment analysis was carried out on the target genes associated with these miRNAs. Subsequently, the survival outcomes of miR-27a across various cancers were analyzed using the Kaplan-Meier Plotter platform. The expression levels and prognostic significance of miR-27a and miR-27a-5p in renal cancer were assessed via the UALCAN and ENCORI databases, respectively. Moreover, the association between miR-27a-5p and LC3A, LC3B, and LC3C was assessed. We conducted a correlation analysis between the genes targeted by miR-27a-5p and autophagy-related genes. The **Supplementary Materials** provided an explanation of the representativeness of the transcriptome samples.

### 2.15 Experimental materials

The 786-O renal carcinoma cells were sustained in RPMI-1640 medium, whereas ACHN cells were maintained in MEM medium. Both culture media were enriched with 10% fetal bovine serum (FBS) and 1% penicillin-streptomycin. The cells were maintained in a humidified incubator at 37 °C with 5% CO_2_, and their morphology and proliferation were routinely observed using a microscope. Experiments were typically conducted when cells reached 70–80% confluence. 18β-Glycyrrhetinic acid (18β-GA; purity > 97%, Cat. No. G10105-10G; Sigma, USA) was dissolved in DMSO to create a 10 mM stock solution for later applications. A complete list of experimental materials is provided in **Table 2**.

**Table 2:** Materials and reagents.

Reagent name	Manufacturer	Item number
Human renal clear cell adenocarcinoma cell 786-O	Shanghai Fuheng Biotechnology Co.Ltd	FH0229
Human renal cell adenocarcinoma cell ACHN	Shanghai Fuheng Biotechnology Co.Ltd	FH0549
Human renal cortex proximal convoluted tubule epithelial cells HK-2	Wuhan Punosai Life Technology Co.Ltd	CL-0109
MEM/EBSS medium	Hyclone USA	Cat.No.SH30024.01
RPMI-1640 medium	Gibco USA	Cat.No.8122261
Fetal bovine serum	Gemini USA	Cat.No.900-108
Trypsin-EDTA solution	Solarbio Beijing	Cat.No.T1320
Penicillin-streptomycin solution	Gibco USA	Cat.No.P1400
PBS phosphate buffer	Hyclone USA	Cat.No.SH30256.01
Trizol	Thermo Fisher Scientific USA	Cat.No.343706
TB Green ^®^ Premix Ex Taq™ II	TaKaRa Japan	Cat.No.RR820A
PrimeScript™ RT Reagent Kit	TaKaRa Japan	Cat.No.RR047A
4% paraformaldehyde	Shanghai Sangon Biotechnology Co.Ltd.	Cat.No.D16013
Total protein extraction kit	Jiangsu KeyGEN BioTECH Co.Ltd.	Cat.No.KGP2100
BCA Quantitative Kit	Jiangsu Beyotime Biotechnology Co.Ltd.	Cat.No.P0012
Crystal violet	Beijing Biotopped Technology Co.Ltd.	Cat.No.C6470-5g
Cell apoptosis kit	Jiangsu KeyGEN BioTECH Co.Ltd.	Cat.No.KGA1023
Cell cycle kit	Jiangsu KeyGEN BioTECH Co.Ltd.	Cat.No.KGA511
PVDF membrane	Millipore USA	Cat.No.ISEQ00010
Protein-free rapid blocking solution	Enzyme Biotechnology Co.Ltd.	Cat.No.PS108P
GAPDH antibody	Wuhan Sanying Biotechnology Co.Ltd.	Cat No. 60004-1-Ig
LC3 rabbit polyclonal antibody	Wuhan Sanying Biotechnology Co.Ltd.	Cat No. 14600-1-AP
ECL chemiluminescent solution	Affinity USA	Cat.No.KF8003
Goat anti-mouse	Affinity USA	Cat.No.S0002
Goat anti-rabbit	Affinity USA	Cat.No.S0001
SDS-PAG loading buffer	Kangwei Century Biotechnology Co.Ltd.	Cat.No.CW0027
Electrophoresis buffer (10×)	Enzyme Biotechnology Co.Ltd.	Cat.No.PS105S
PAGE gel preparation reagent (10%)	Enzyme Biotechnology Co.Ltd.	Cat.No.PG212
Protein-free rapid blocking solution	Enzyme Biotechnology Co.Ltd.	Cat.No.PS108P
TBST	Wuhan Selleck Biotechnology Co.Ltd.	Cat.No.G0001

### 2.16 CCK-8 assay

Renal carcinoma cells were plated into 96-well plates at a density of 8,000 cells per well, while HK-2 cells were seeded at 6,000 cells per well. After incubation at 37 °C for 24 hours, various concentrations of 18β-GA and 5-fluorouracil (5-Fu) were applied to the wells. The cells were then cultured for additional time periods of 24, 48, or 72 hours. Cell viability was assessed using the CCK-8 assay kit; following the addition of the reagent, plates were incubated at 37 °C for 1 hour. Absorbance values at 450 nm were subsequently recorded with a microplate spectrophotometer. All experiments were performed in triplicate to ensure reproducibility.

### 2.17 Lentiviral transfection

Well-conditioned renal cancer cells were digested and counted, and the cell density was set to 4×10^4^ cells/mL. Subsequently, cells were seeded into six-well plates at a volume of 2 mL culture medium per well and incubated for 24 hours. Once cell confluence reached approximately 30%, the initial medium was discarded and substituted with 1 mL of new medium. Lentiviral vectors encoding GFP-tagged miR-27a-5p knockdown, miR-27a-5p overexpression, or corresponding negative control (empty vector), provided by Shanghai Genechem, were then added. The lentivirus volume was determined according to the multiplicity of infection (MOI) and viral titer using the formula: virus volume = (MOI × cell number)/titer. For infection enhancement, 100 μL of HiTransG A was added to ACHN cells, while 100 μL of HiTransG P was added to 786-O cells. The plates were gently mixed and placed in a 37 °C incubator. After 10 hours of transduction, the medium was changed to 2 mL of fresh complete medium to sustain further culture. Transfection efficiency was monitored at 24, 48, and 72 hours post-transduction under a fluorescence microscope by assessing GFP signal in both bright-field and GFP-specific channels. Once transfection efficiency exceeded 90%, downstream experiments were initiated.

### 2.18 Clone formation assay

Two cell lines were seeded into 6-well plates at a density of 500 cells per well, with three replicate wells per experimental group. Cells were treated according to their respective experimental conditions and cultured with medium refreshed every three days. After 10 days, the experiment was concluded. Cells were rinsed with PBS, fixed for 30 minutes using 4% paraformaldehyde, and subsequently rinsed again with PBS to eliminate the fixative. Subsequently, 2 mL of 2% crystal violet solution was added to each well for staining at room temperature over 15 minutes. Following two PBS washes, the plates were air-dried before imaging. Colonies with diameters exceeding 1 mm were counted as individual clones. The entire experiment was independently repeated three times.

### 2.19 Cell cycle detection

Well-growing renal cancer cells were adjusted to an appropriate concentration and inoculated into 60 mm culture dishes. After complete adhesion, starvation treatment was performed to bring the cell cycle of each group to the same level. Subsequently, cells were processed based on their assigned experimental conditions. Following a 24-hour incubation period, they were harvested after trypsinization, washed using PBS, and then fixed overnight at 4 °C with 70% precooled ethanol. The fixative was removed by PBS washing, followed by the addition of 500 μL of freshly prepared cell cycle staining solution (Rnase A:PI = 1:9). The cells were kept at room temperature in the dark for a 30-minute incubation period, after which cell cycle distribution was analyzed using flow cytometry. This procedure was performed in triplicate to ensure reproducibility.

### 2.20 Apoptosis detection

Well-growing renal cancer cells were inoculated in 6-well plates. Subsequently, the cells were subjected to treatments as defined by their respective experimental groups. On the following day, the cell supernatant and cells were harvested, washed twice with PBS, and the supernatant was discarded. Then, 500 μL of Binding Buffer was added and thoroughly mixed. After adding the staining solution and ensuring complete mixing, the samples were kept at room temperature in the dark for a 15-minute incubation period. Apoptosis analysis was performed by flow cytometry within one hour. The experiment was conducted in triplicate to ensure consistency and reliability of the results.

### 2.21 qRT-PCR experiment

Renal cancer cells were collected in a sterile and enzyme-free tube, washed with PBS, and then 1 mL Trizol was added and mixed well by pipetting. After ice bath, 200 μL chloroform was added and mixed well. The mixture was kept on ice for 10 minutes and then centrifuged. The upper layer was transferred to a new tube, and an equal volume of isopropanol was added and gently mixed. After ice bath and centrifugation, the supernatant was discarded, and 70% ethanol was added and mixed well. After centrifugation, the supernatant was discarded and the precipitate was air-dried. The precipitate was dissolved in ddH2O. 1 μL of RNA sample was taken to measure the OD value and concentration. An OD260/OD280 ratio of 1.8-2.1 was considered as qualified purity. cDNA reverse transcription was performed using PrimeScript™ RT reagent Kit with gDNA Eraser. Each group had 3 replicates. Data were analyzed by the 2^-△△CT^ method.

### 2.22 Western blotting for protein immunodetection

Protein levels in the samples were determined using a BCA protein assay kit. After separation by SDS-PAGE and transfer to PVDF membranes, proteins were fixed onto the membrane surface. The membranes were subsequently blocked with a rapid blocking solution for 15 minutes, followed by overnight incubation at 4 °C with specific primary antibodies. Following extensive washing, the membranes were incubated with corresponding secondary antibodies at room temperature for 2 hours. The PVDF membranes were immersed in the ECL working solution for 2 minutes and exposed using a chemiluminescence imaging system. The protein bands were quantified for protein expression using Image J.

### 2.23 Statistical analysis

All quantitative data are presented as mean ± standard deviation, based on at least three independent replicates. Statistical evaluations were conducted using GraphPad Prism 10. Group comparisons were conducted via one-way ANOVA. In cases of homogenous variances, ANOVA was applied; for heterogenous variances, Tamhane’s T2 test was used. A p value below 0.05 was considered statistically significant.

## 3 Result

### 3.1 The targets of 18β-GA and renal cancer were predicted

**Figure 2:**
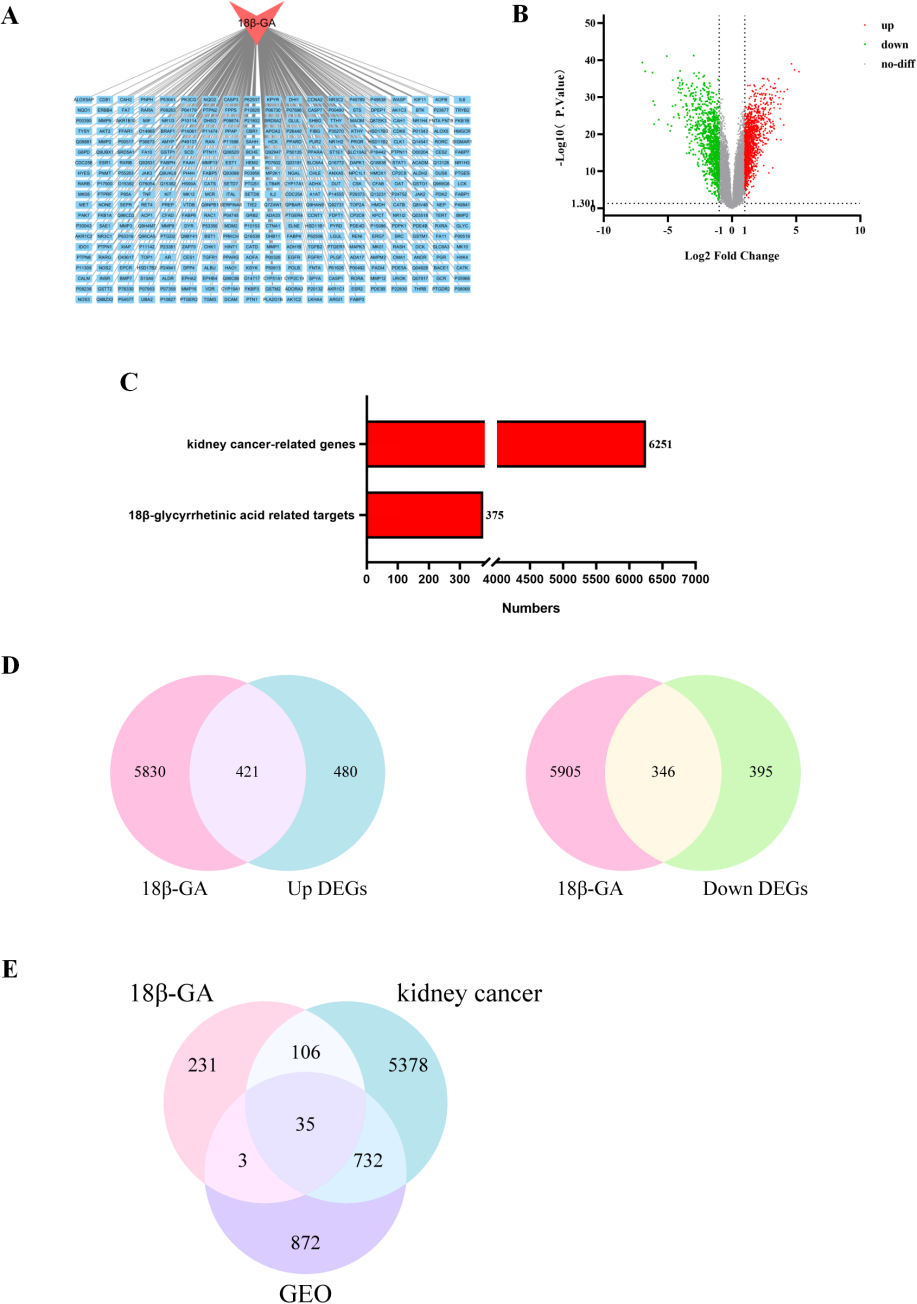
The genes of the 18β-GA, renal carcinoma genes and differentially expressed renal carcinoma genes. **(A)** Network diagram of 18β-GA-targets. Red represents the 18β-GA and blue represents the targets of 18β-GA. **(B)** Volcano plot of differentially expressed genes of GSE46699. Green represents down-regulated genes, red represents up-regulated genes, and gray represents no difference or significance. **(C)** All genes of renal carcinoma and the number of targets of 18β-GA. **(D)** Intersection of upper and lower regulatory genes and 18β-GA targets in renal carcinoma. Pink indicates the target gene of 18β-GA, while blue and green indicate up-regulated and down-regulated genes, respectively. **(E)** Intersection diagram of 18β-GA and renal carcinoma targets. Pink represents genes for 18β-GA, purple represents differential genes from GEO database, and blue represents genes for renal carcinoma.

Network pharmacology predicted 375 targets for 18β-GA (**Figure 2A**). The GEO dataset GSE46699 of renal cancer chips was analyzed, and 1322 up-regulated genes and 1045 down-regulated genes were obtained (**Figure 2B**), with 6251 renal cancer genes (**Figure 2C**). Intersecting the up-regulated and down-regulated genes of renal cancer with drug targets yielded 421 and 346 targets, respectively (**Figure 2D**). Next, the intersection of 18β-GA, renal cancer and differentially expressed genes was taken, and a total of 35 targets were obtained (**Figure 2E**).

### 3.2 Results of WGCNA analysis

As shown in **Supplementary Figure 1A**, the power value used in this analysis result is 24. Using the chosen power value, genes are categorized into 11 modules, excluding the grey module, which lacks reference significance, as depicted in **Supplementary Figures 1B, C**. Pearson correlation analysis was performed to determine the correlation coefficients and corresponding p-values between module characteristic genes and traits. Modules associated with traits were identified using a cutoff of |correlation coefficient| ≥ 0.3 and p < 0.05. As illustrated in **Supplementary Figure 1D**, the turquoise, black, and grey60 modules exhibiting the highest correlation strengths were selected for further analysis. The intersection of these genes and the previous intersection genes is taken again to obtain 20 more important targets (**Supplementary Figure 1E**). The heatmap data indicates that about half of these 20 targets are highly expressed in renal cancer (**Supplementary Figure 1F**). **Supplementary Figure 1G** shows the top ten up-regulated and down-regulated genes. PCA is applied to the intersection targets to assess sample representativeness, as illustrated in **Supplementary Figure 1H**. The distinct separation between the tumor and normal groups suggests significant sample representativeness.

### 3.3 Enrichment analysis

To further explore the key targets of 18β-GA in treating renal cancer, we used Cytoscape software to screen 20 targets. As shown in **Supplementary Figure 2A**, we identified STAT1, KIT, HMOX1, CASP1, HCK, FABP1 and IDO1 as key targets. **Supplementary Figure 2B** illustrates the use of the Degree value to assess each target’s contribution within the topological map. We continued to analyze their correlations, as shown in **Supplementary Figure 2C**. Most of the targets showed positive correlations, while FABP1 and KIT tended to show negative correlations. GO analysis indicated that the 20 targets were enriched in BP terms associated with inflammatory responses, CC terms suggesting localization in the cytoplasm and extracellular space, and MF terms linked to functions like protein tyrosine kinase activity (**Supplementary Figure 2D**). KEGG analysis found that these 20 targets were enriched in three signaling pathways, including “Pathways in cancer”, “PPAR signaling pathway” and “Aldosterone-regulated sodium reabsorption” (**Supplementary Figure 2E**).

### 3.4 Machine learning algorithms

**Figure 3:**
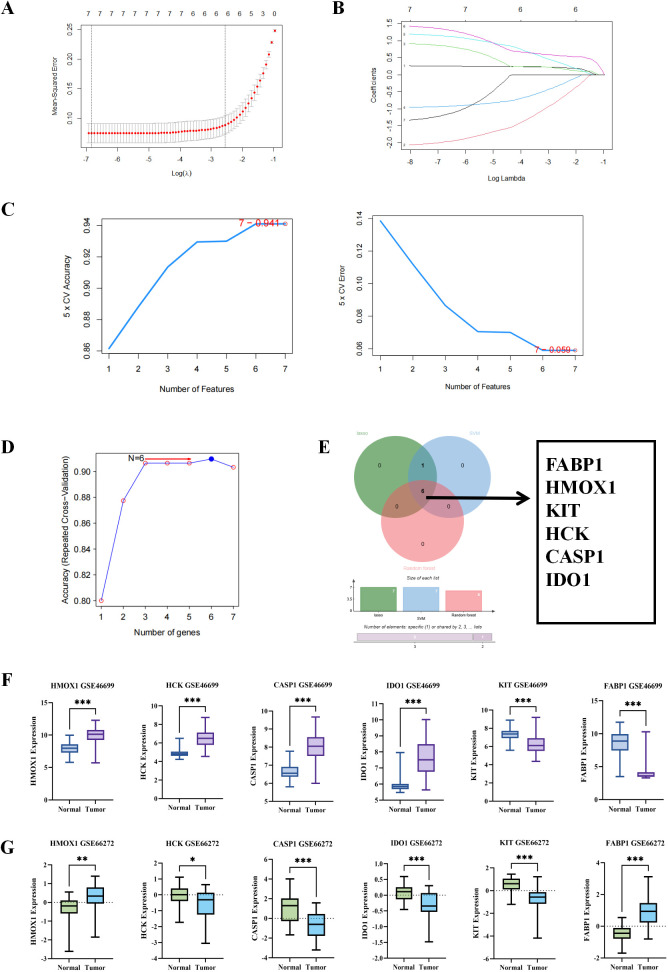
Machine learning algorithms analyze core targets. **(A)** The change process of the optimal penalty coefficient λ in Lasso regression model. **(B)** Seven genes were identified by Lasso regression algorithm. **(C)** Seven genes were identified by SVM-RFE algorithm. **(D)** Six genes were selected by the random forest algorithm. **(E)** Lasso algorithm, SVM algorithm and random forest algorithm were intersected to obtain 6 intersection genes, which we identified as the core targets. **(F, G)** Validation of the validation set and training set of machine learning algorithms. *p < 0.05, **p < 0.01, ***p < 0.001, indicate that the results are statistically significant.

We conducted Lasso analysis on the 7 key targets obtained from the previous step and found that all of them were significant under this algorithm (**Figures 3A, B**). As shown in **Figure 3C**, the SVM algorithm also indicated that these targets were significant. As depicted in **Figure 3D**, the random forest algorithm revealed that the accuracy reached its peak when 6 genes were included. Subsequently, we took the intersection of the genes obtained from the three algorithms and identified FABP1, HMOX1, KIT, HCK, CASP1, and IDO1 as the core targets, as illustrated in **Figure 3E**. Next, we verified the results obtained from the machine learning algorithms. We found that these targets exhibited significant expression differences in the dataset GSE46699. Using the GSE66272 dataset as the validation set revealed significant differences in these targets between the tumor and normal groups. Therefore, we concluded that the genes obtained through the machine learning algorithms were reliable, as shown in **Figures 3F, G**. Additionally, we observed that the genes HMOX1, HCK, CASP1, and IDO1 were all related to autophagy. Thus, we hypothesized that there might be a correlation between drug treatment for renal cancer and autophagy. We chose these four genes and the autophagy-related genes MAP1LC3A (LC3I) and MAP1LC3B (LC3II) as primary targets to explor.

### 3.5 Clinical relevance analysis

**Figure 4:**
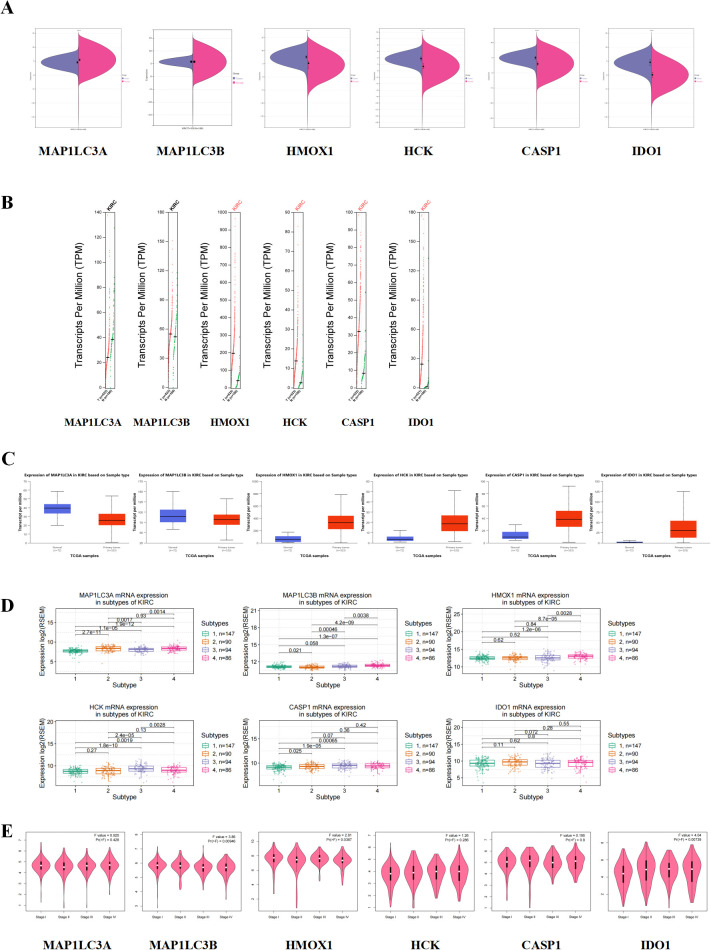
Clinical correlation analysis of Hub gene. **(A)** mRNA expression level of Hub gene. Purple is the tumor group, pink is the normal group. **(B)** Hub gene copy number expression level. Red represents the tumor group. **(C)** Hub gene protein expression level. **(D)** The expression level of Hub gene in renal carcinoma subtypes. **(E)** Relationship between Hub gene and clinical stage of renal carcinoma.

Core target expression was examined at both transcriptional and translational levels. At the mRNA level, MAP1LC3A showed high expression in normal tissues, whereas the other targets were predominantly expressed in renal cancer tissues, with MAP1LC3B showed no significant difference in expression between normal and renal cancer tissues (**Figure 4A**). In copy number analysis, HMOX1, HCK, CASP1, and IDO1 were significantly elevated in renal cancer patients (**Figure 4B**). At the protein level, HMOX1, HCK, CASP1, and IDO1 were elevated in renal cancer tissues, while MAP1LC3A and MAP1LC3B were highly expressed in the normal group (**Figure 4C**). Renal cancer staging indicated that MAP1LC3A was highly expressed in stage 2 renal cancer and HCK was highly expressed in stage 3 (**Figure 4D**). Analysis of core targets in renal cancer clinical staging revealed that MAP1LC3B and HMOX1 levels were elevated in the early stage, whereas IDO1 significantly increased in the middle and late stages (**Figure 4E**).

### 3.6 Immunohistochemical and survival prognosis results of core targets

Immunohistochemical analysis revealed that MAP1LC3B exhibited high expression in normal tissues, whereas other genes showed elevated expression in renal cancer tissues (**Supplementary Figure 3A**). Immunofluorescence localization showed that MAP1LC3A and MAP1LC3B were expressed in centriolar satellites and primary cilia, HMOX1 was expressed in the Golgi apparatus and plasma membrane, HCK was expressed in vesicles, plasma membrane and cytoplasm, CASP1 was expressed in the cytoplasm and nucleoplasm, and IDO1 was expressed in microtubules and primary cilia (**Supplementary Figure 3B**). Elevated levels of MAP1LC3B and HMOX1 were significantly associated with better prognosis and higher survival rates in renal cancer patients, whereas other targets showed no significant impact (**Supplementary Figure 3C**).

### 3.7 The relationship between mutations of core targets and renal cancer

**Figure 5:**
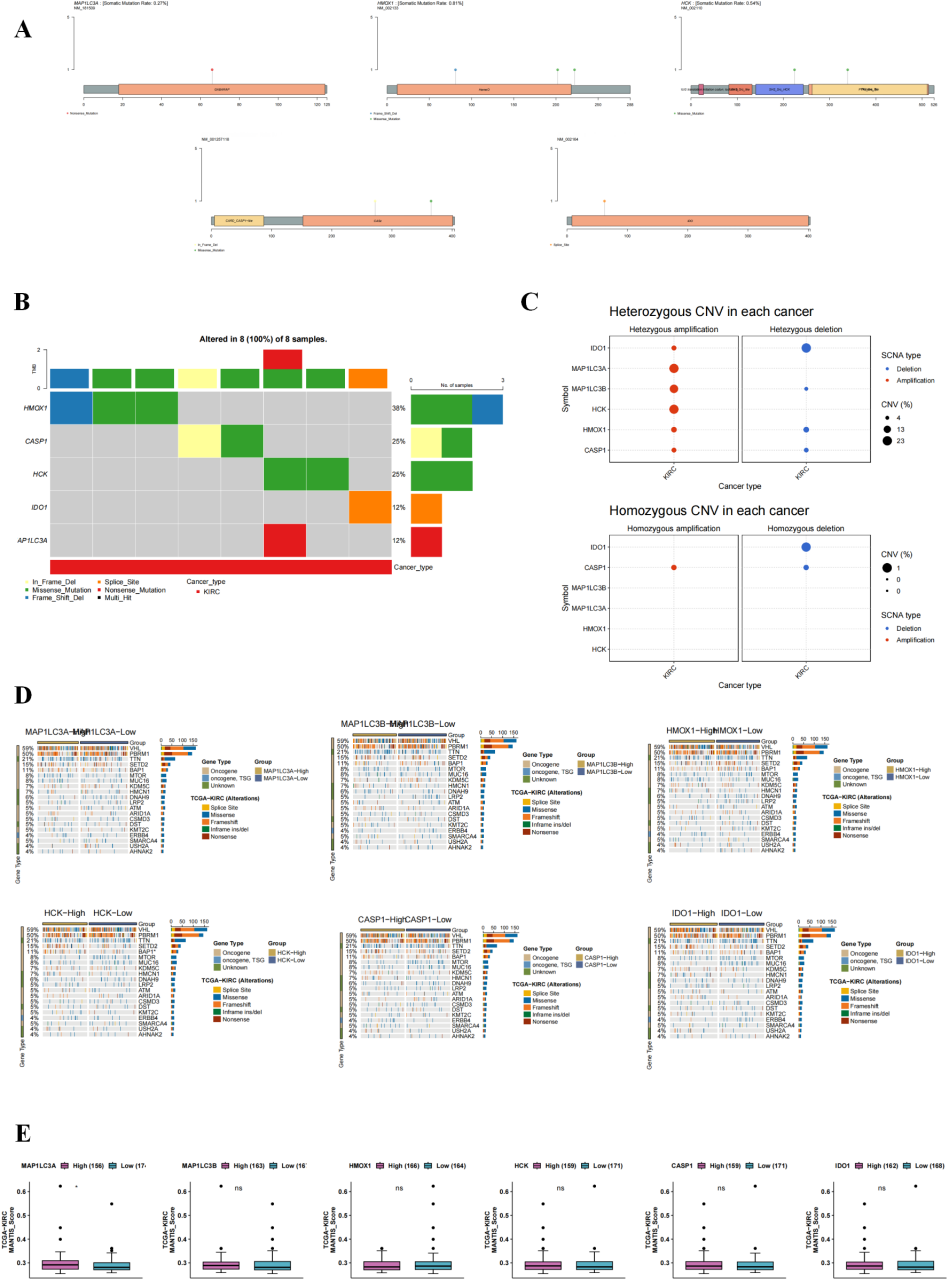
Effect of Hub gene mutation on renal carcinoma. **(A)** SNV mutation site of the core target. The circle color indicates the mutation type, and the line length indicates the mutation frequency. **(B)** Waterfall diagram of SNV mutation frequency of Hub gene. The bar chart above shows the proportion of Hub gene mutations in 8 samples. **(C)** Bubble maps of heterozygous and pure heterozygous CNV mutations in Hub genes. The larger the bubble, the higher the proportion of mutations. **(D)** Association between driver genes and core gene mutations. **(E)** Box pattern of MSI expression level of Hub gene.

The occurrence of tumors is characterized by genomic alterations. Therefore, we analyzed whether the core targets were changed at the genomic level through SNV and CNV mutation analysis. We found that the correlation between these genes and mutations was not strong (**Figure 5A**). Then, we further analyzed the frequency of harmful mutations in Hub genes. As shown in **Figure 5B**, the mutation frequency of HMOX1 was the highest. CNV includes heterozygous mutations and homozygous mutations, among which homozygous mutations will induce more severe disease outcomes. As shown in **Figure 5C**, we found that the main mutation form of these core targets was amplification. As shown in **Figure 5D**, the high mutation of these genes was most closely related to VHL and PBRM1, and high expression could promote the expression of proto-oncogenes. Microsatellite instability (MSI) is characterized by alterations in the length of microsatellite sequences due to insertions or deletions during DNA replication, typically resulting from defects in mismatch repair (MMR) functions. **Figure 5E’s** box plot of hub gene MSI expression levels reveals that only MAP1LC3A positively correlates with MSI, suggesting its elevated expression is linked to increased microsatellite instability.

### 3.8 The relationship between methylation of core targets and renal cancer

**Figure 6:**
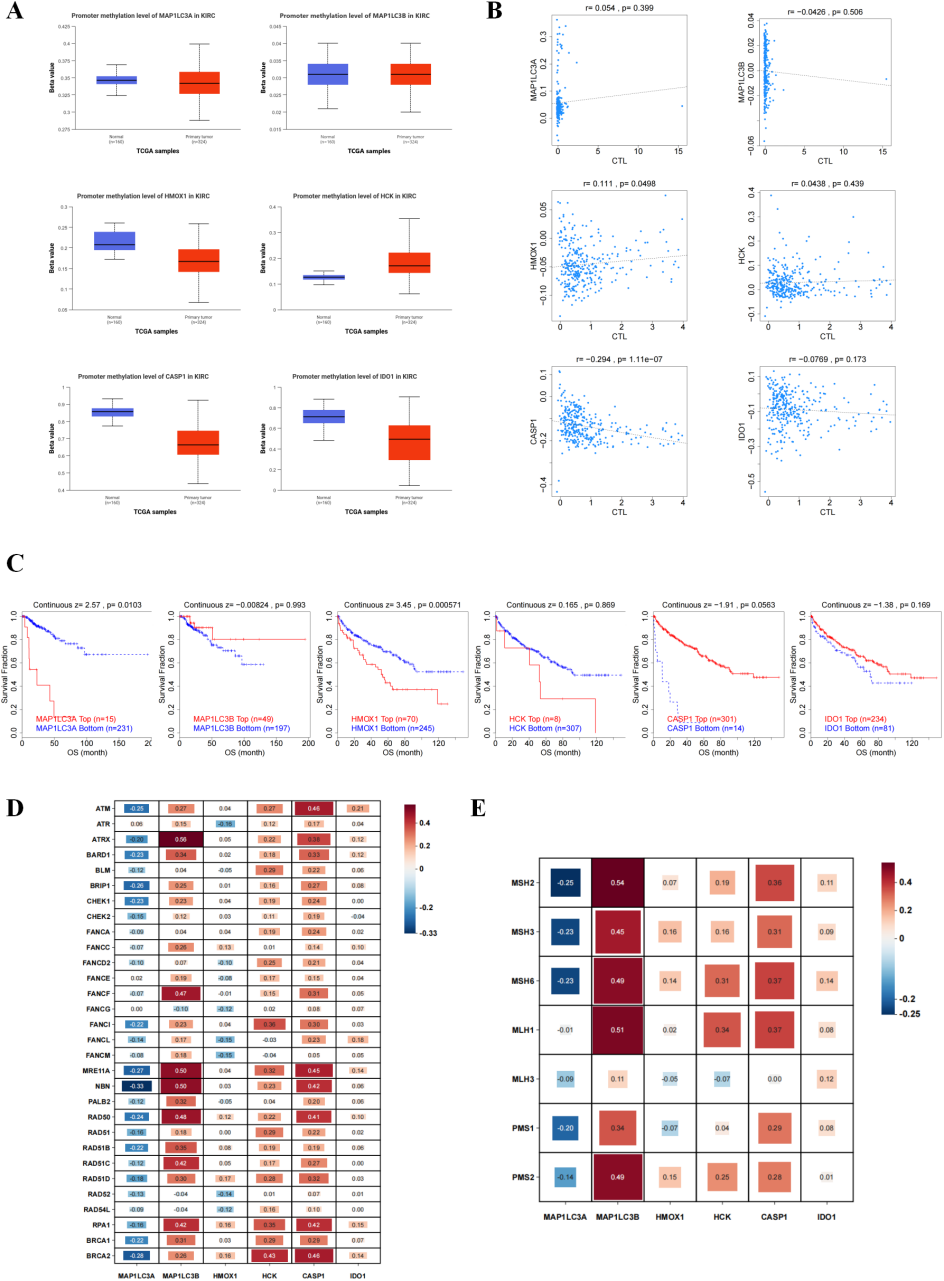
Hub gene is involved in epigenetic regulation and repair of damaged genes. **(A)** Map of methylation expression level of Hub gene. Blue and red represent normal and tumor groups, respectively. **(B)** Correlation between the methylation level of Hub gene and CTL markers. CTL are cytotoxic T cells. **(C)** Draw survival curves of hypermethylated and hypomethylated subpopulations of Hub gene. **(D, E)** Heatmap of correlation between Hub genes and HRR and MMR repair systems. Red color represents positive correlation and blue color represents negative correlation. The size of the box represents the magnitude of the correlation; the larger the box, the stronger the correlation.

As shown in **Figure 6A**, after methylation, HCK is highly expressed in thes renal cancer group, whiles the expression of MAP1LC3B shows no significant difference. The other genes are all at low expression levels. Additionally, as shown in **Figure 6B**, HMOX1 is positively correlated with cytotoxic T cells, while CASP1 is negatively correlated. The other targets have no significant correlation. As shown in **Figure 6C**, after gene methylation, MAP1LC3A and HMOX1 are negatively correlated with survival risk, meaning that highly methylated genes are unfavorable for patient survival. We examined the association between mutations in core targets and the DNA repair machinery. The vertical axis represents repair system-related genes, and the horizontal axis represents Hub genes. We found that MAP1LC3A shows a significant negative correlation with two repair systems, while MAP1LC3B, HCK, CASP1, and IDO1 show a positive correlation. Among them, the correlation of MAP1LC3B is the strongest (**Figures 6D, E**).

### 3.9 The association between immune responses and renal carcinoma

The tumor microenvironment is composed of various cells, which can both promote and inhibit tumor development, being complex and variable. Through relevant scoring, we can gain multi-dimensional insights and thus more accurately grasp this delicate environment. Among them, StromalScore is used to assess the proportion of stromal components in renal cancer tissues, ImmuneScore reflects the proportion of immune cells, and ESTIMATEScore reveals tumor purity. **Supplementary Figure 4A** shows that HMOX1, HCK, CASP1 and IDO1 have significant positive correlations with renal cancer scores, while MAP1LC3A and MAP1LC3B have negative correlations. The single-cell analysis of clear cell renal cell carcinoma (KIRC, dataset GSE121636) primarily illustrates the distribution of immune cell subpopulations within the tumor microenvironment and the expression of key targets in these cells, as depicted in **Supplementary Figure 4B**. MAP1LC3A is highly expressed in CD8T cells, MAP1LC3B and CASP1 are expressed in various immune cells, HMOX1 and HCK are expressed in monocytes/macrophages, and IDO1 is expressed in dendritic cells. Meanwhile, immune checkpoint analysis indicates that the targets show a positive correlation trend with immune checkpoint molecules (**Supplementary Figure 4C**). Further, five analysis algorithms were used to analyze the correlations of core targets in immune cells, as shown in **Supplementary Figure 4D**. MAP1LC3A is negatively correlated with these immune cells, while HMOX1, HCK and CASP1 are positively correlated.

### 3.10 The association between core targets and antitumor drug therapy

As shown in **Supplementary Figure 5A**, HMOX1 is closely associated with multiple drugs and targets, while other targets have relatively fewer related drugs. There is no information on drug treatment for MAP1LC3A and MAP1LC3B. IFN-γ has antiviral, antitumor, and immunomodulatory effects, and TGF-β can exert tumor-suppressive effects in early-stage tumors. **Supplementary Figure 5B** illustrates that HCK, CASP1, and IDO1 show a positive correlation with IFN-γ expression, whereas MAP1LC3A exhibits a negative correlation. Additionally, HCK is positively correlated with TGF-β.GDSC and CTRP are used to screen the sensitivity of antitumor drugs. As illustrated in **Supplementary Figure 5C**, in CTRP and GDSC, MAP1LC3A, MAP1LC3B, HMOX1, and IDO1 are positively correlated with drug sensitivity, while CASP1 and HCK are negatively correlated with drug sensitivity.

### 3.11 Molecular docking analysis of 18β-GA with core target proteins

**Figure 7:**
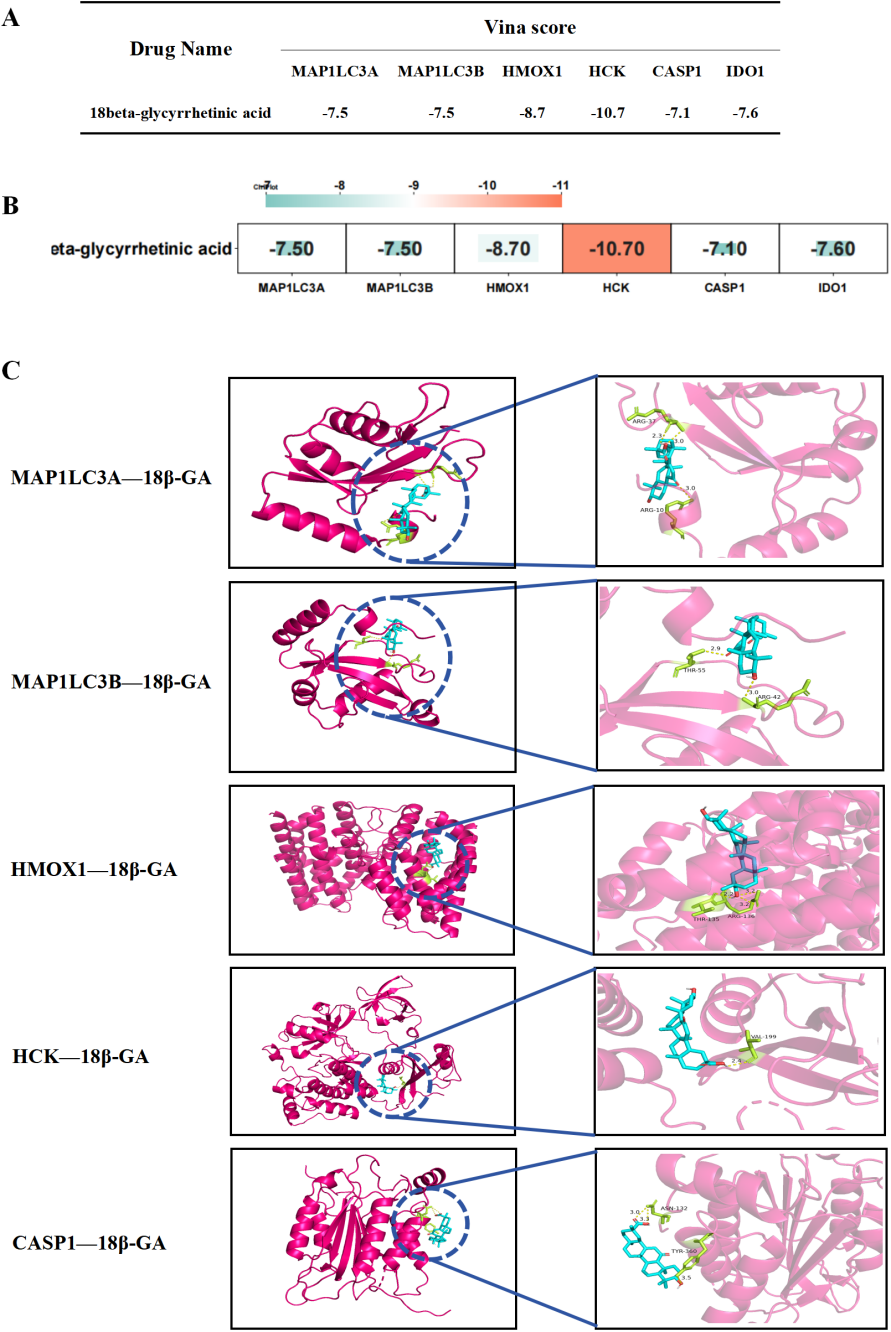
Molecular docking of the core target with the 18β-GA. **(A, B)** Molecular docking binding energy of the core target with the 18β-GA. **(C)** Molecular docking visualization of the core target and the 18β-GA.

Molecular docking simulations are employed to forecast the interaction affinity between drugs and their target molecules, effectively simulating the drug absorption and metabolic processes that occur in the human body. The binding energy of the core target 18β-GA was predicted through molecular docking, as shown in **Figures 7A, B**, with the binding energy being less than -7 kcal/mol, indicating a good binding ability. The result was visualized as shown in **Figure 7C**.

### 3.12 Toxicity analysis of active ingredients

As shown in **Supplementary Figure 6A**, the toxicity grade of 18β-GA is 4, indicating relatively low toxicity. As depicted in **Supplementary Figures 6B, C**, the toxicity prediction results are presented. The radar plot displays the confidence level of positive toxicity outcomes relative to the mean toxicity of structurally analogous compounds. In each toxicity response, the closer the blue dot is to the center of the circle, the lower the toxicity; conversely, the farther it is, the higher the toxicity. As shown in **Supplementary Figure 6D**, the toxicity dose, toxicity grade, and affected organs of 18β-GA are summarized. Overall, its toxicity is relatively low, mainly affecting the respiratory system and the heart.

### 3.13 Prediction of upstream transcription factors and downstream target proteins

**Figure 8:**
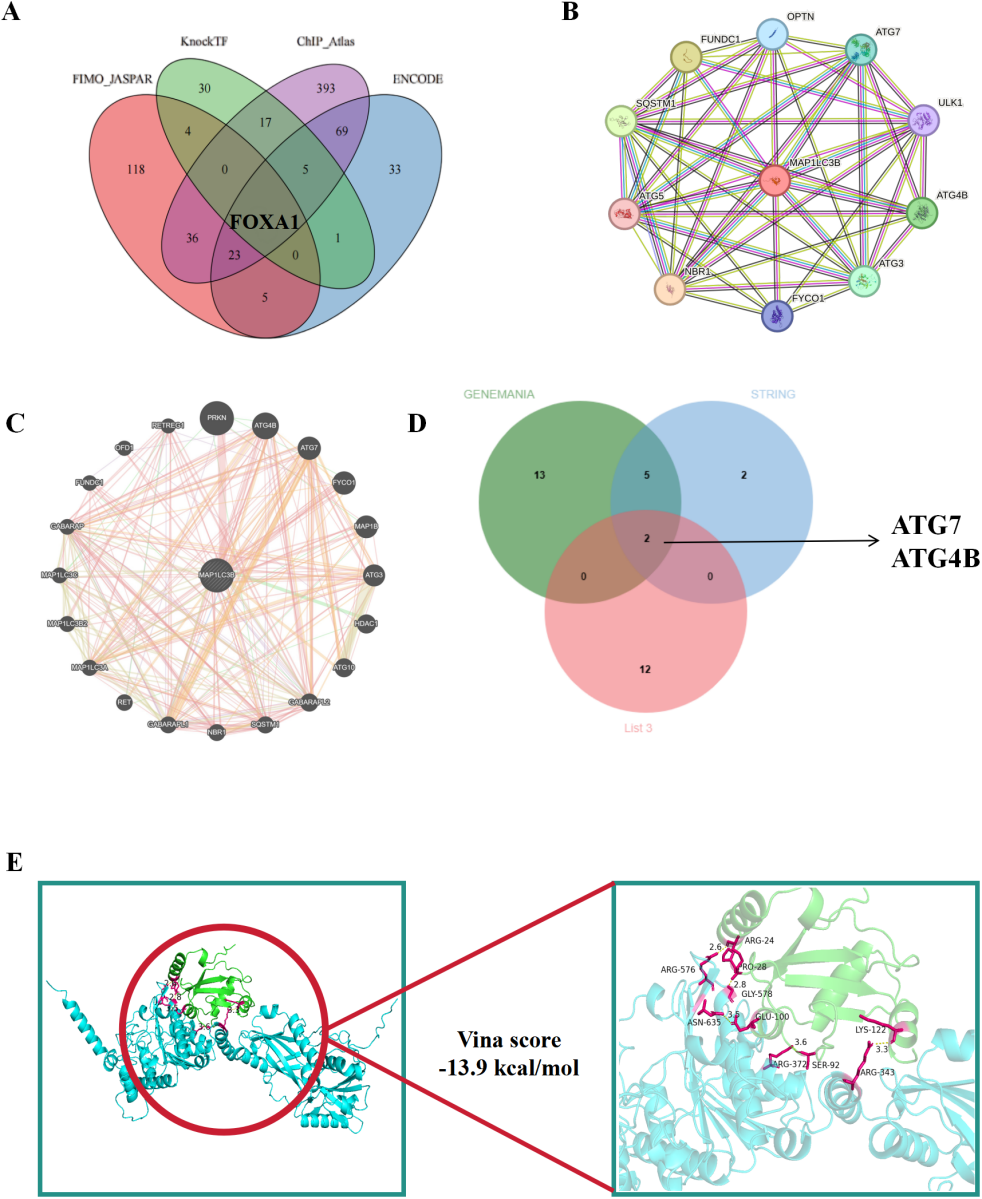
Upstream transcription factor and downstream target protein screening. **(A)** Screening of upstream transcription factors of the core target MAP1LC3B. **(B, C)** Query of downstream target protein of core target MAP1LC3B. **(D)** Three database intersection genes of downstream target proteins. **(E)** Molecular docking visualization of MAP1LC3B and ATG7.

Since the core targets are mostly related to autophagy, and the MAP1LC3B protein, due to its direct anchoring to autophagosomes and strong association with the core steps of autophagy, has become a key indicator for measuring autophagic activity, we chose to study MAP1LC3B. As shown in **Figure 8A**, the upstream transcription factor of MAP1LC3B was identified as FOXA1; the downstream target proteins were ATG7 and ATG4B (**Figures 8B–D**). Based on the analysis of binding energy through molecular docking, the interaction energy between MAP1LC3B and ATG7 was calculated to be -13.9 kcal/mol, and with ATG4B was -11.1 kcal/mol. Therefore, ATG7 with the higher binding energy was selected and visualized (as shown in **Figure 8E**).

### 3.14 Total transcriptome sequencing analysis

**Figure 9:**
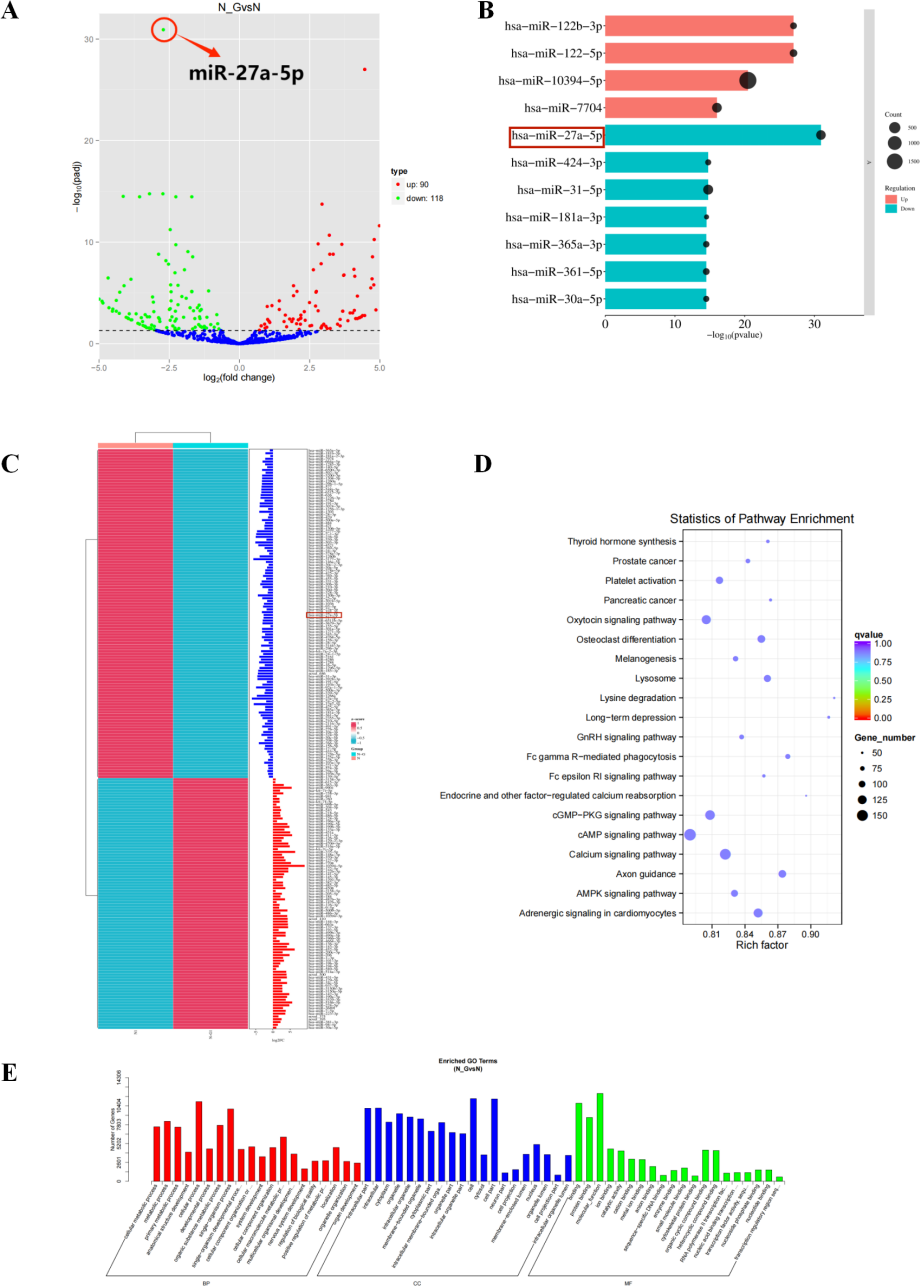
Total transcriptome sequencing and identification of miR-27a. **(A)** Volcano plot of differential expression analysis. Each dot represents a miRNA, with blue dots indicating miRNAs without significant differences, red dots indicating up-regulated miRNAs with significant differences, and green dots indicating down-regulated miRNAs with significant differences. **(B)** Bar chart of the top ten differentially expressed miRNAs ranked by p-value. **(C)** Hierarchical clustering heatmap of differentially expressed miRNAs. Red indicates high expression and blue indicates low expression. **(D)** Scatter plot of enriched pathways. The y-axis represents the pathway name, the x-axis represents the rich factor (the proportion of candidate genes in the background gene set), the size of the dots indicates the number of differentially expressed genes in this pathway, and the color of the dots corresponds to different q-value ranges. **(E)** GO Enrichment Analysis Plot of Related Genes.

The results of the total transcriptome sequencing showed that miR-27a-5p had the most significant difference in renal cancer after 18β-GA intervention, and its expression was down-regulated after drug intervention (**Figures 9A–C**). Functional enrichment analysis of predicted target genes regulated by miR-27a-5p using GO and KEGG revealed significant involvement in pathways such as cAMP and AMPK signaling (**Figure 9D**), and the biological processes were related to cell metabolism, etc (**Figure 9E**). miR-27a exhibited elevated expression levels in renal cancer (**Supplementary Figure 7A**), which negatively impacted the prognosis and survival outcomes of patients with this condition (**Supplementary Figure 7B**). The pan-cancer analysis showed that higher expression levels were negatively correlated with patient prognosis and overall survival (**Supplementary Figure 7C**). miR-27a-5p exhibited elevated expression levels in renal cancer (**Supplementary Figure 7D**), which positively influenced the prognosis and survival rates of patients with this condition (**Supplementary Figure 7E**). miR-27a-5p was negatively correlated with autophagy-related proteins LC3A and LC3C, and positively correlated with LC3B (**Supplementary Figure 7F**). **Supplementary Figure 7G** indicated that the genes targeted by miR-27a-5p were mostly positively correlated with autophagy-related genes. The **Supplementary Materials** (**Supplementary Figure 8**) demonstrated the accuracy and reliability of the sequencing data.

### 3.15 18β-GA suppresses renal cancer cell proliferation

#### 3.15.1 Impact of 18β-GA on renal cancer cell viability

**Figure 10:**
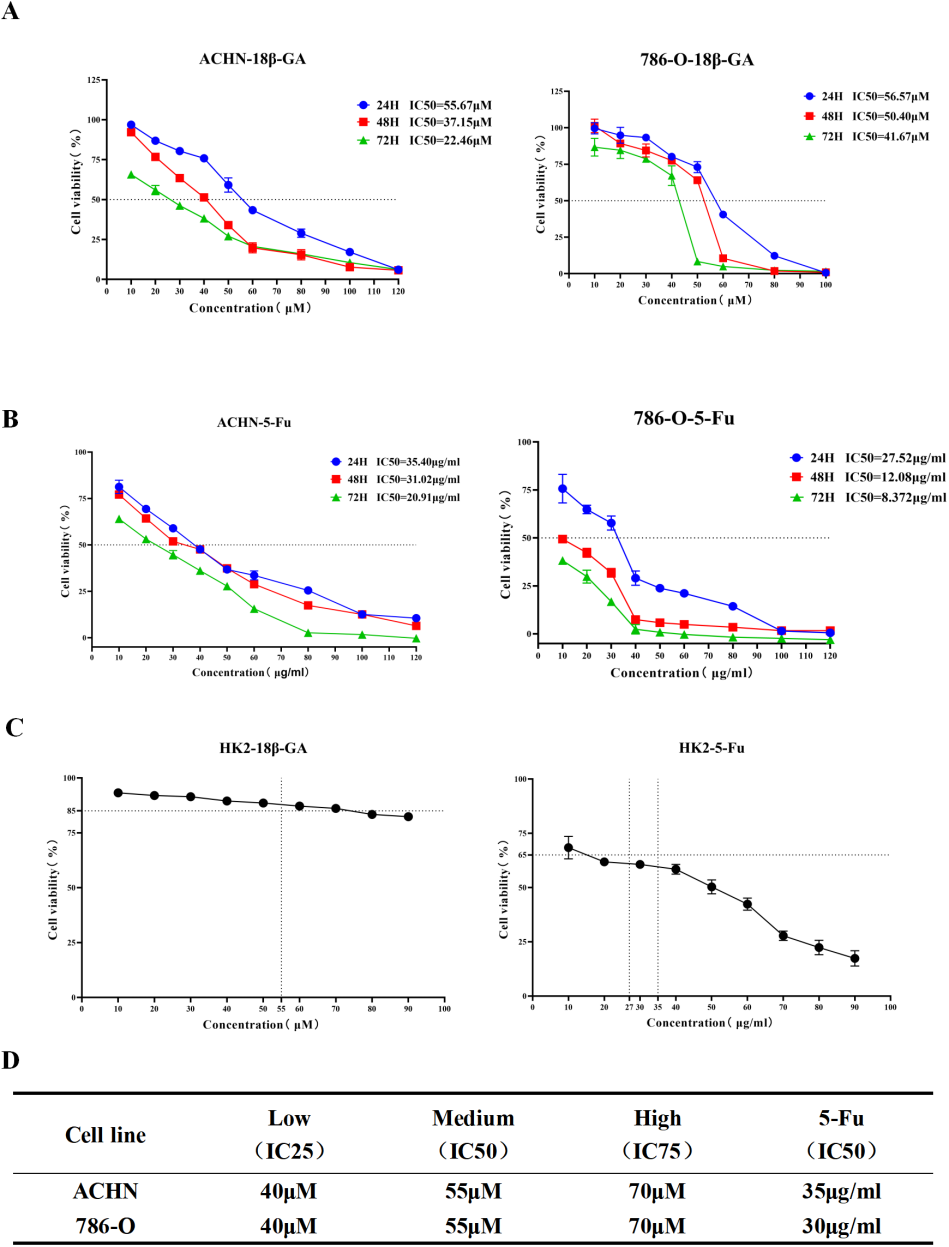
18β-GA inhibits the survival rate of renal cancer cells. **(A)** ACHN and 786-O renal cancer cell lines were treated with different concentrations of 18β-GA for 24, 48 and 72 hours, and cell viability was determined by the CCK-8 method. **(B)** ACHN and 786-O renal cancer cell lines were treated with different concentrations of 5-Fu for 24, 48 and 72 hours, and cell viability was determined by the CCK-8 method. **(C)** Effects of 18β-GA and the positive control drug 5-Fu on normal renal cells HK-2. **(D)** The experimental time was set at 24 hours. The concentrations of 18β-GA corresponding to IC75, IC50 and IC25 for ACHN and 786-O cells, and the IC50 concentration of the positive drug 5-Fu for the two renal cancer cell lines.

Treatment of renal cancer cells with 18β-GA resulted in a concentration-dependent reduction in cell viability, as demonstrated by CCK-8 assays (**Figure 10A**). Similarly, the positive control agent 5-Fu displayed a dose-responsive suppression of cell proliferation (**Figure 10B**). Following a 24-hour exposure, the low, medium, and high treatment concentrations were set at 40, 55, and 70 μM for both cell lines, with 5-Fu exhibiting IC50 values of 35 μg/mL and 30 μg/mL, respectively (**Figure 10D**). Evaluation of cytotoxicity revealed that HK-2 normal renal cells maintained over 85% viability when exposed to 70 μM 18β-GA (P < 0.05), whereas treatment with 27 μg/mL of 5-Fu reduced normal cell viability to below 65%. These findings suggest that 18β-GA potently suppresses renal carcinoma cell growth while exerting significantly lower toxicity on normal kidney cells compared to 5-Fu. Based on these results, a 24-hour treatment duration was chosen for subsequent experiments (**Figure 10C**).

#### 3.15.2 18β-GA induces apoptosis in renal cancer cells

**Figure 11:**
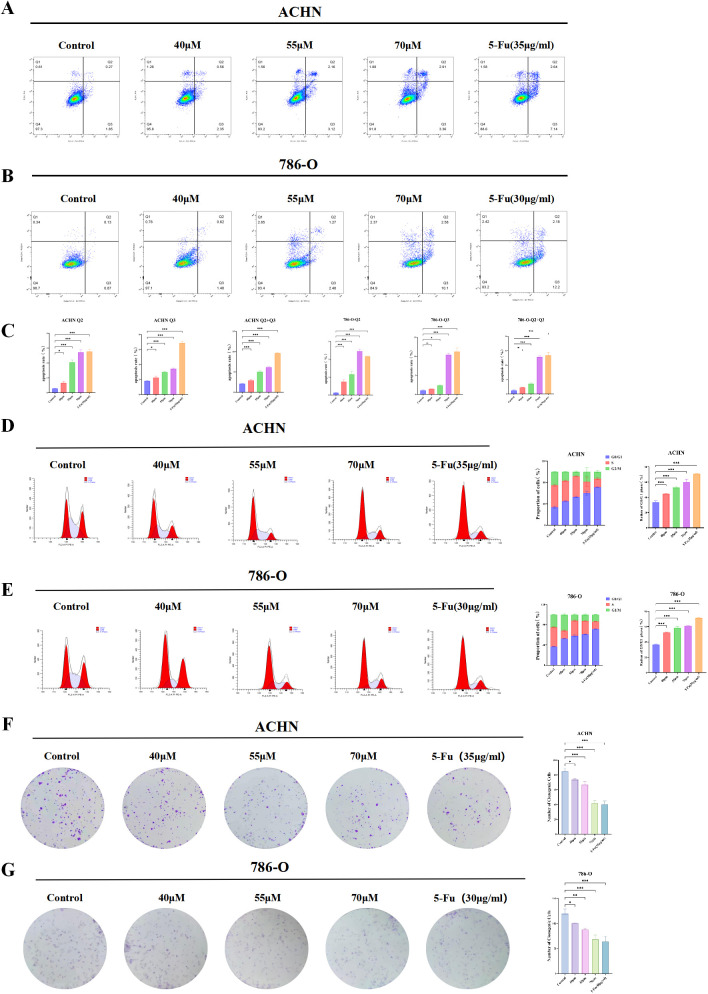
Effects of 18β-GA on the Proliferation Capacity of Renal Cancer Cells. **(A-C)** Detection of the effects of low, medium and high concentrations of 18β-GA and the positive drug 5-Fu on the apoptosis rate of renal cancer cells. **(D, E)** Detection of the effects of low, medium and high concentrations of 18β-GA and the positive drug 5-Fu on the cell cycle of renal cancer cells. **(F, G)** Detection of the effects of low, medium and high concentrations of 18β-GA and the positive drug 5-Fu on the clone formation capacity of renal cancer cells. *p < 0.05, **p < 0.01, ***p < 0.001, indicate that the results are statistically significant.

Following 24 hours of treatment, the apoptosis rate in renal cancer cells rose progressively with increasing concentrations of 18β-GA. In ACHN cells, both 18β-GA and 5-Fu treatments across all concentration levels induced significantly higher apoptosis compared to the control group (P < 0.05, **Figure 11A**). Similarly, in 786-O cells, early apoptosis rates were markedly elevated after exposure to 18β-GA or 5-Fu relative to the control (P < 0.05, **Figure 11B**), although the late apoptosis rate in the low-dose 18β-GA group did not differ significantly from the control (**Figure 11C**). These findings demonstrate that 18β-GA effectively enhances apoptotic cell death in renal carcinoma cells.

#### 3.15.3 18β-GA arrests the cell cycle of renal cancer cells

The experimental results demonstrated that both ACHN and 786-O cells experienced cell cycle arrest at the G0/G1 phase. After 24 hours of drug exposure, a marked and dose-dependent rise in the percentage of cells halted in the G0/G1 phase was observed relative to the control group (P < 0.05) (**Figures 11D, E**). The findings suggest that 18β-GA suppresses renal cancer cell proliferation by inducing G0/G1 phase cell cycle arrest.

#### 3.15.4 18β-GA suppresses the clonal proliferation of renal cancer cells

Cell counting was analyzed using ImageJ software. The results showed a concentration-dependent decrease in the colony-forming ability of ACHN cells relative to the control group, with reductions diminishing as concentration increased. The positive drug 5-Fu exhibited a similar trend (P < 0.05) (**Figure 11F**). The clonogenic ability of 786-O cells was reduced at low, medium, and high drug concentrations, with a significant reduction observed as the concentration increased (P < 0.05) (**Figure 11G**). The results demonstrated that 18β-GA suppressed the clonogenic potential of renal cancer cells in a dose-responsive manner.

### 3.16 miR-27a-5p modulates autophagy to suppress renal cancer cell proliferation

#### 3.16.1 The transfection efficiency of miR-27a-5p lentivirus

**Figure 12:**
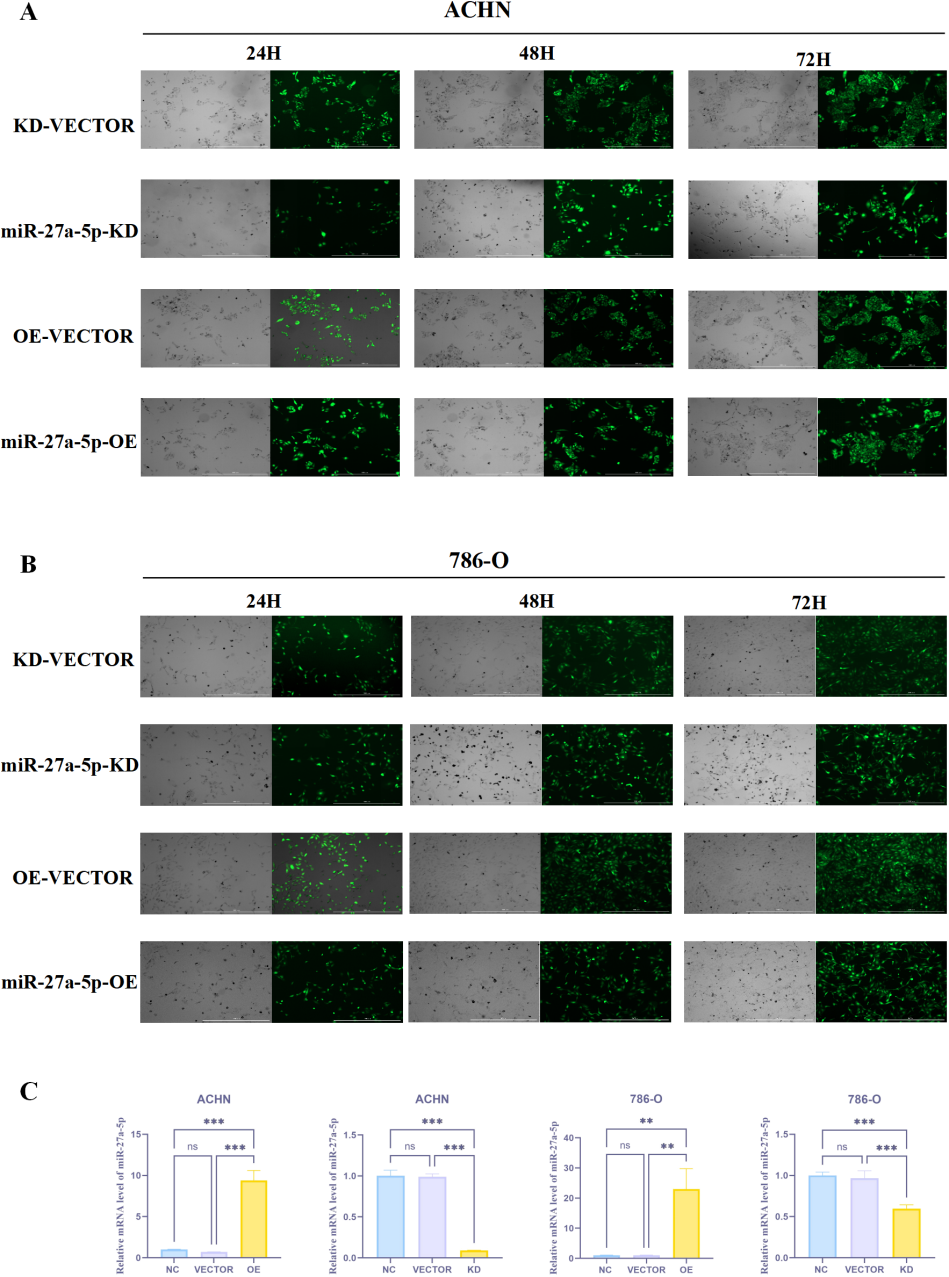
The efficiency of miR-27a-5p transfection in human renal cancer cells. **(A)** Fluorescence of transfection efficiency of human renal cancer cell ACHN. **(B)** Fluorescence of transfection efficiency of human renal cell carcinoma 786-O. **(C)** Statistical results of miR-27a-5p mRNA expression in ACHN and 786-O cells after lentivirus transfection. **p < 0.01, ***p < 0.001, indicate that the results are statistically significant.

Images captured at 24, 48, and 72 hours post-transduction revealed efficient lentiviral infection in both ACHN and 786-O cell lines (**Figures 12A, B**). qRT-PCR analysis demonstrated no significant difference in miR-27a-5p expression levels between the Vector-KD and Vector-OE control groups (p > 0.05), confirming the suitability of the viral vector system. In contrast, miR-27a-5p expression was markedly downregulated in the miR-27a-5p KD group compared to the Vector-KD group (p < 0.05), while it was significantly upregulated in the miR-27a-5p OE group relative to the Vector-OE group (p < 0.001) (**Figure 12C**), thereby verifying successful lentiviral transfection.

#### 3.16.2 Effect of miR-27a-5p on colony-forming ability

**Figure 13:**
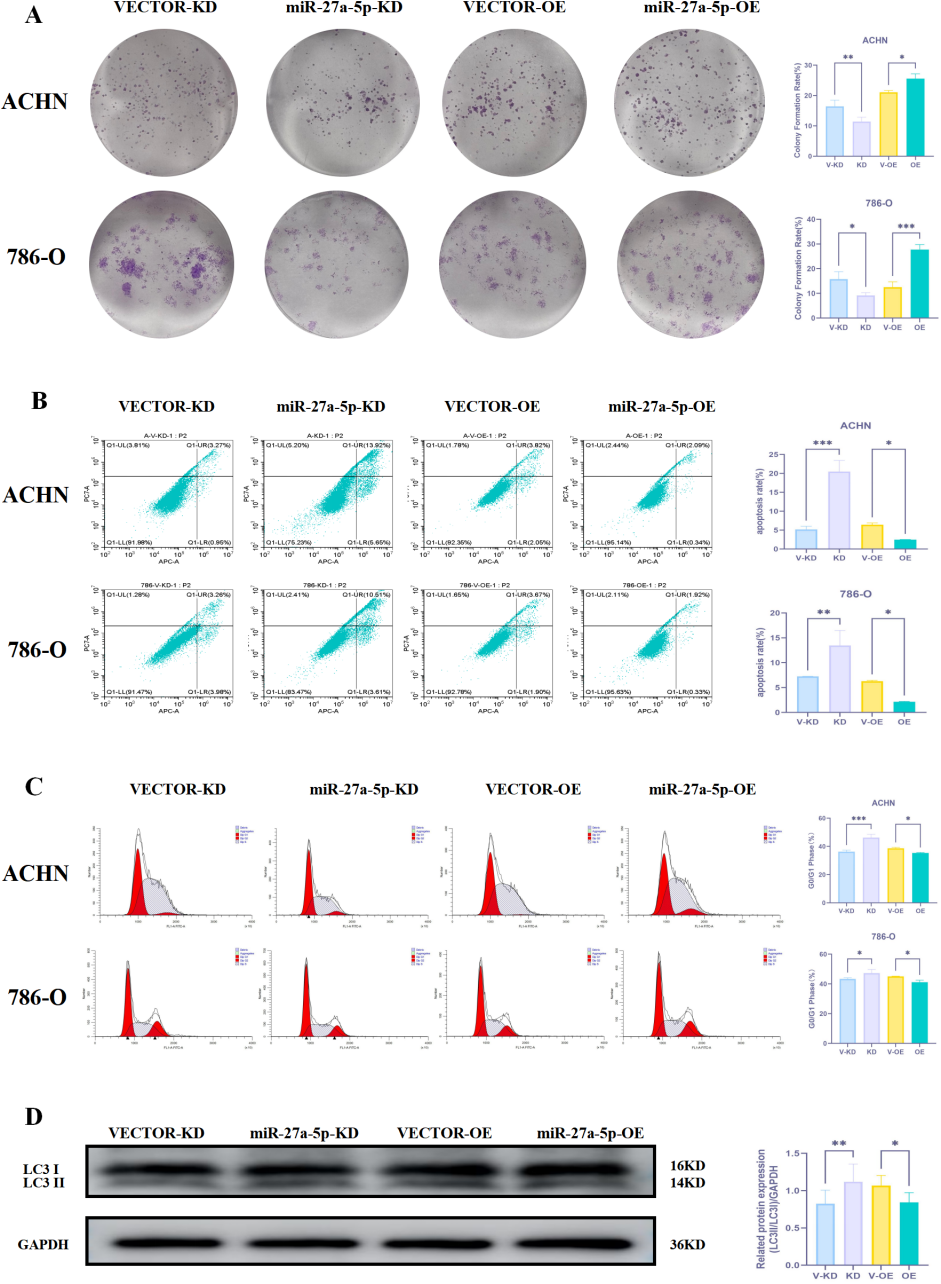
The effect of miR-27a-5p on the proliferation phenotype of human renal cancer cells. **(A)** The effect of miR-27a-5p on the clone formation ability of human renal cancer cells. **(B)** The effect of miR-27a-5p on the apoptosis of human renal cancer cells. **(C)** The effect of miR-27a-5p on human renal cancer cell cycle. **(D)** The effect of miR-27a-5p on the expression of LC3 protein in human renal cancer cells *p < 0.05, **p < 0.01, ***p < 0.001 indicate that the results are statistically significant.

The clone formation assay assessed the impact of miR-27a-5p on the clonogenic capacity of human renal cancer cells. The experimental findings indicated that in ACHN and 786-O, the miR-27a-5p KD group exhibited a significant reduction in crystal violet positive staining compared to the Vector-KD group (p < 0.05). Conversely, the miR-27a-5p OE group showed a significant increase in crystal violet positive staining relative to the Vector-OE group (p < 0.05) (**Figure 13A**). The study found that miR-27a-5p knockdown reduced, whereas its overexpression enhanced, the clonogenic potential of renal cancer cells.

#### 3.16.3 Influence of miR-27a-5p on apoptotic activity

Apoptosis was assessed using flow cytometry. In the human renal cancer cell line ACHN, the apoptosis rates for the Vector-KD, miR-27a-5p KD, Vector-OE, and miR-27a-5p OE groups were 5.19 ± 0.85%, 20.46 ± 2.99%, 6.43 ± 0.48%, and 2.47 ± 0.06%, respectively. In the 786-O cell line, the corresponding values were 7.23 ± 0.04%, 13.46 ± 3.03%, 6.27 ± 0.19%, and 2.15 ± 0.09%. In both ACHN and 786-O cells, silencing miR-27a-5p significantly increased apoptosis compared to the respective vector control (p < 0.001), indicating that miR-27a-5p knockdown promotes programmed cell death in renal cancer cells. Conversely, overexpression of miR-27a-5p led to a significant reduction in apoptosis relative to the Vector-OE group in both cell lines (p < 0.05) (**Figure 13B**), suggesting that elevated miR-27a-5p levels suppress apoptotic activity.

#### 3.16.4 Effect of miR-27a-5p on cell cycle progression

Cell cycle analysis for each cell group was conducted using flow cytometry. The study found that in ACHN and 786-O cells, the miR-27a-5p KD group had a significantly higher percentage of cells in the G0/G1 phase compared to the Vector-KD group (p < 0.001), while the miR-27a-5p OE group had a lower percentage than the Vector-OE group (p < 0.05) (**Figure 13C**). The findings demonstrated that miR-27a-5p knockdown arrests renal cancer cells in the G0/G1 phase.

#### 3.16.5 Effect of miR-27a-5p on LC3 protein expression

The effect of miR-27a-5p on LC3 protein expression was evaluated in human renal cancer cell lines ACHN and 786-O by assessing LC3 protein levels across various experimental groups. Western blot results demonstrated that LC3II protein expression was significantly increased in the miR-27a-5p KD group compared to the Vector-KD group (p < 0.01), indicating autophagy activation. Conversely, LC3II expression was significantly decreased in the miR-27a-5p OE group compared to the Vector-OE group (p < 0.05), suggesting autophagy inhibition (**Figure 13D**). These results indicate that miR-27a-5p knockdown influences autophagy through LC3 regulation, leading to inhibited proliferation of renal cancer cells.

### 3.17 18β-GA suppresses renal cancer cell proliferation by modulating autophagy via the miR-27a-5p/LC3 pathway

#### 3.17.1 Impact of 18β-GA on miR-27a-5p’s regulation of renal cancer clonogenicity

**Figure 14:**
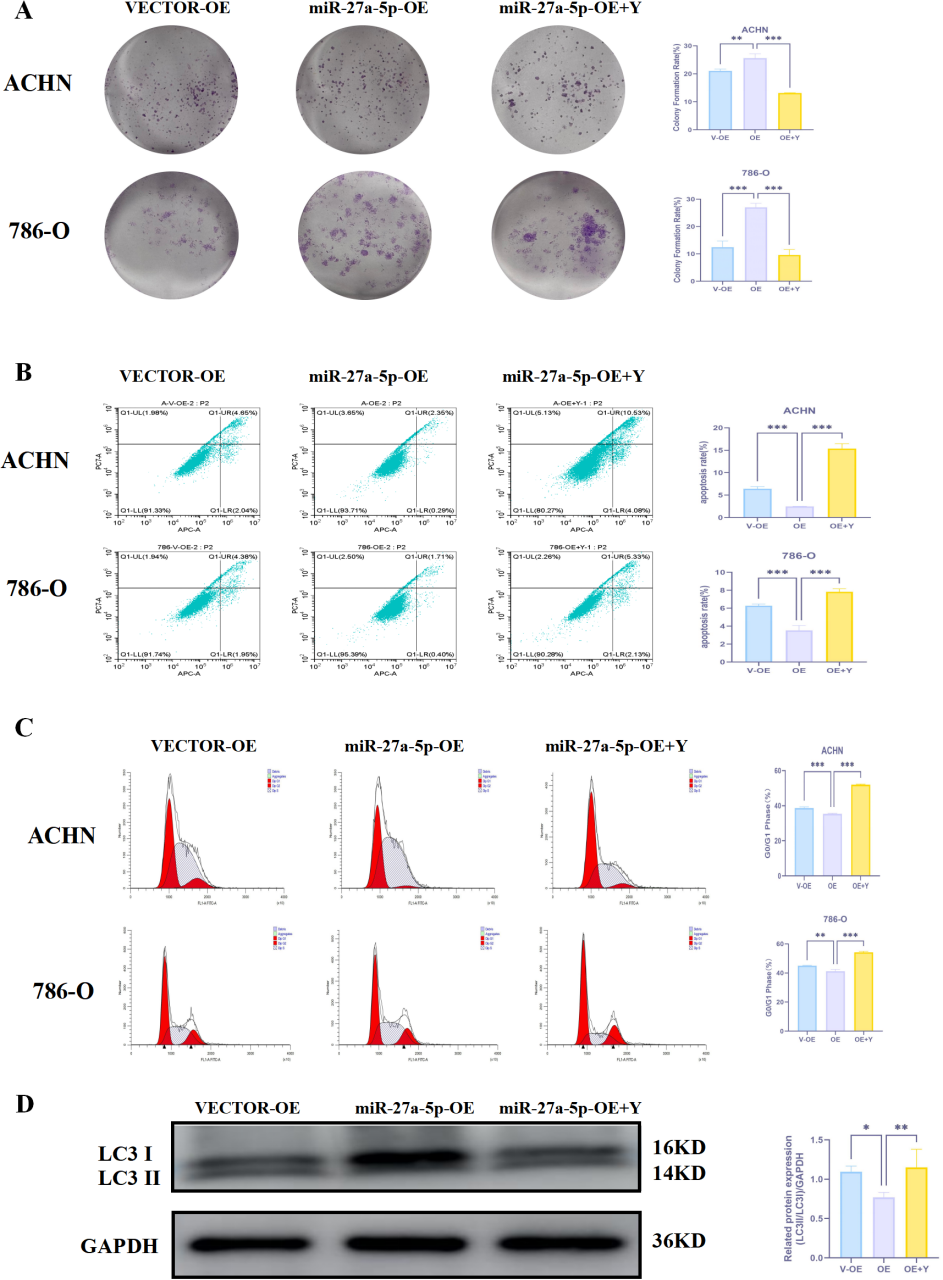
18-GA regulates the effect of miR-27a-5p on the proliferation phenotype of human renal cancer cells. **(A)** 18-GA regulates the effect of miR-27a-5p on the clone formation ability of human renal cancer cells. **(B)** 18-GA regulates the effect of miR-27a-5p on the apoptosis of human renal cancer cells. **(C)** 18-GA regulates the effect of miR-27a-5p on the cell cycle of human renal cancer cells. **(D)** 18-GA regulates the effect of miR-27a-5p on the expression of LC3 protein in human renal cancer cells. *p < 0.05, **p < 0.01, ***p < 0.001, *p < 0.05, **p < 0.01, ***p < 0.001 indicate that the results are statistically significant.

The clone formation assay assessed the impact of 18β-GA on the clone formation ability of human renal cancer cells through the regulation of miR-27a-5p. Experimental results indicated that in ACHN and 786-O human renal cancer cells, the miR-27a-5p OE group exhibited a significant increase in positive crystal violet staining compared to the Vector-OE group (p < 0.01). However, the miR-27a-5p OE + Y group showed a significant decrease in staining compared to the miR-27a-5p OE group (p < 0.001) (**Figure 14A**). The findings demonstrated that 18β-GA suppresses renal cancer cell clone formation via miR-27a-5p.

#### 3.17.2 The impact of 18β-GA on miR-27a-5p-mediated apoptosis regulation in renal cancer cells

In ACHN, apoptosis rates were 6.43 ± 0.48% for the Vector-OE group, 2.47 ± 0.06% for the miR-27a-5p OE group, and 15.37 ± 1.08% for the miR-27a-5p OE+Y group. In the 786-O cell line, the rates were 6.27 ± 0.19%, 3.52 ± 0.55%, and 7.82 ± 0.35%, respectively. In ACHN and 786-O, the miR-27a-5p OE group exhibited a significantly reduced apoptosis rate compared to the Vector-OE group (p < 0.001). The miR-27a-5p OE+Y group exhibited a significantly higher apoptosis rate compared to the miR-27a-5p OE group (p < 0.001). The findings demonstrated that 18β-GA facilitates apoptosis in renal cancer cells via miR-27a-5p (**Figure 14B**).

#### 3.17.3 The impact of 18β-GA on the renal cancer cell cycle via miR-27a-5p regulation

Cell cycle analysis for each cell group was conducted using flow cytometry. The study found that in the miR-27a-5p OE group had a lower percentage of cells in the G0/G1 phase compared to the Vector-OE group (p < 0.05). Additionally, the miR-27a-5p OE + Y group exhibited a higher percentage of cells in the G0/G1 phase than the miR-27a-5p OE group (p < 0.001) (**Figure 14C**). The findings demonstrated that 18β-GA can halt renal cancer cell progression at the G0/G1 phase via miR-27a-5p.

#### 3.17.4 The effect of 18β-GA on LC3 protein through regulating miR-27a-5p

We examined the impact of 18β-GA on LC3 protein expression in human renal cancer cells ACHN and 786-O by measuring LC3 levels in each cell group. Western blot analysis showed that LC3II protein expression decreased in the miR-27a-5p OE group compared to the Vector-OE group in ACHN and 786-O renal cancer cells (p < 0.05), and increased in the miR-27a-5p OE + Y group compared to the miR-27a-5p OE group (p < 0.01) (**Figure 14D**). The findings indicate that 18β-GA may suppress renal cancer cell proliferation by modulating LC3 via miR-27a-5p.

## 4 Discussion

**Figure 15:**
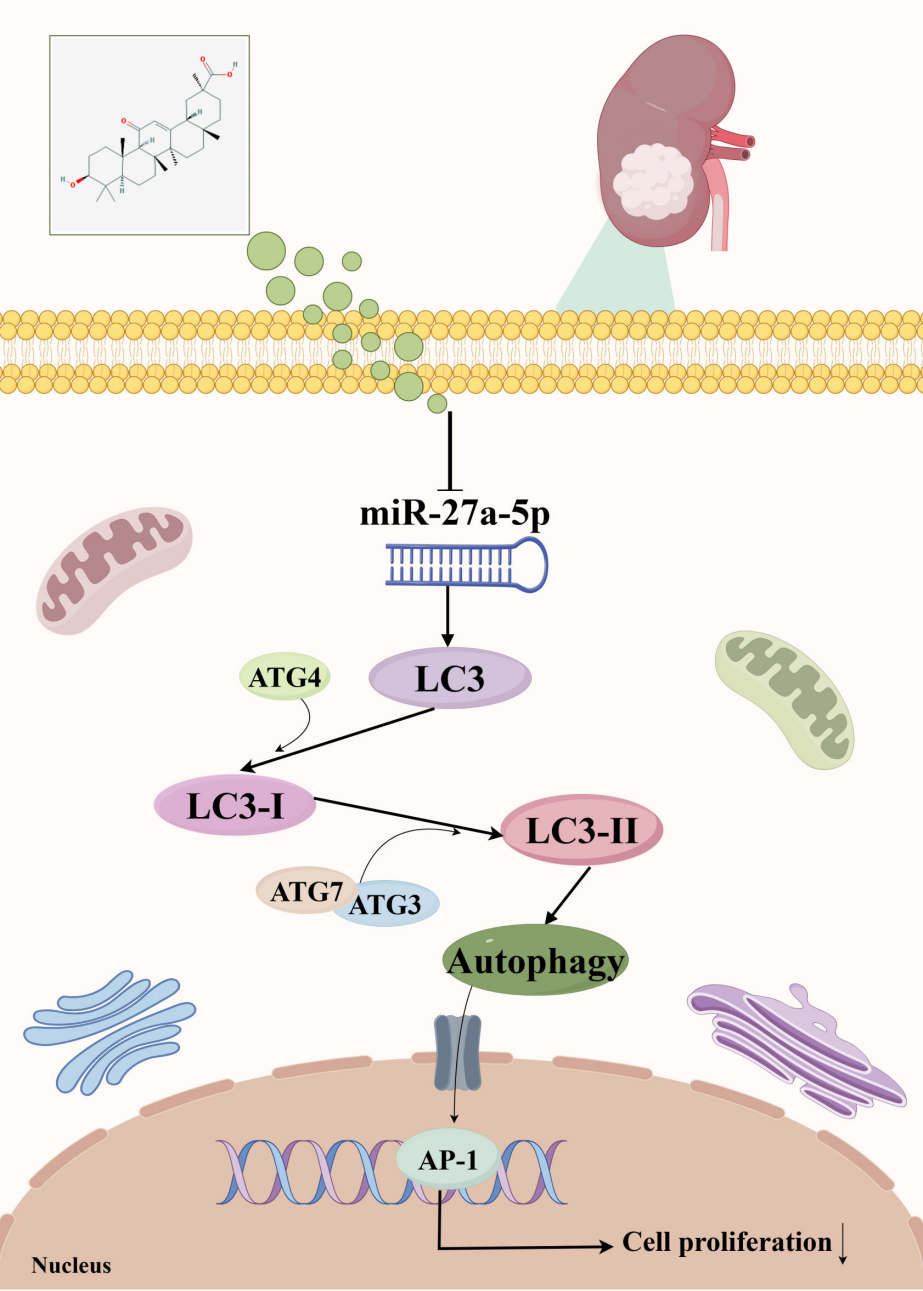
Graphic abstract.

Renal cancer is the most fatal among common urinary system cancers (46). Currently, surgical treatment is the main approach, although cytokine therapy is effective for some patients, it has significant toxicity (47). Traditional Chinese medicine not only treats diseases but also provides preventive and health care benefits. It has become increasingly significant in the management of renal carcinoma, offering anticancer effects and enhancing therapeutic efficacy while minimizing toxicity. Building on this foundation, the present study investigates the underlying mechanism through which 18β-glycyrrhetinic acid suppresses the proliferation of kidney cancer cells. A full transcriptome analysis identified the key molecule miR-27a-5p. Network pharmacology and bioinformatics analysis predicted that it is highly expressed in kidney cancer and is related to patient prognosis. We predicted the key targets of 18β-GA in treating kidney cancer as FABP1, HMOX1, KIT, HCK, CASP1, and IDO1. Among them, HMOX1 (48), HCK (49), CASP1 (50) and IDO1 (51) have certain correlations with autophagy and are highly expressed in kidney cancer. The results of KEGG analysis also indicate that the core targets are related to autophagy. In addition, our prediction results show that MAP1LC3B is in a low expression state in kidney cancer tissues, which not only has a positive effect on the survival prognosis of kidney cancer patients but also helps the two DNA repair systems, HHR and MMR, to function better. To further explore the relationship between 18β-GA treatment of kidney cancer and autophagy, we verified the effect on cell proliferation through cycle experiments, apoptosis experiments, and monoclonal experiments. We also verified the regulation of miR-27a-5p on the proliferation of kidney cancer cells through lentivirus transfection technology. By detecting the protein expression of LC3, we further confirmed that 18β-GA induces autophagy through miR-27a-5p/LC3 and inhibits the proliferation of kidney cancer. **Figure 15** presents the graphical abstract.

18β-Glycyrrhetinic acid, a key bioactive constituent of licorice (Glycyrrhiza glabra L.), has emerged as the primary subject of contemporary research, largely due to the limited natural abundance of its 18α isomer (52). Previous research has demonstrated 18β-GA’s significant anti-tumor effects on various human malignancies, and our team has identified its ability to inhibit gastric cancer proliferation through autophagy regulation. In this study, the anti-renal cancer effect of 18β-GA was confirmed through cell experiments. Flow cytometry analysis revealed that the treatment induced apoptosis, caused G0/G1 phase cell cycle arrest, suppressed colony formation, and decreased cell viability in 786-O and ACHN, all of which collectively inhibiting their proliferation. Nevertheless, the precise molecular mechanism underlying these effects requires further investigation. Additionally, the kidney is an important organ for maintaining human balance and excreting metabolic products and drugs (53). Exogenous toxins and drugs may induce nephrotoxicity (54). Research indicates that drugs account for about 20% of nephrotoxicity cases, which can also restrict the use of chemotherapy treatments (55). 18β-GA protects renal function and enhances endogenous antioxidant capacity, thereby mitigating methotrexate-induced renal damage (52), alleviating cisplatin-induced acute nephrotoxicity, and showing promise as a treatment for progressive acute kidney injury (56). It can also activate Nrf2 and PPARγ to protect rats from cyclophosphamide (CP)-induced liver damage (57). GA acts as an inhibitor of P-glycoprotein and multidrug resistance protein, potentially enhancing the efficacy of traditional Chinese medicine by limiting excretion and working synergistically with other components (58). 18β-GA inhibits renal cancer cell proliferation and mitigates drug-induced liver and kidney toxicity, offering protection to these organs. It simultaneously enhances the effectiveness of traditional Chinese medicine in tumor treatment through a synergistic role.

MicroRNA-27a (miR-27a), located on chromosome 19, plays a regulatory role in various cancers, including gastric (59), pancreatic (60), liver (61), kidney (62), prostate (63), breast (64), cervical (65), ovarian (66) and colorectal cancers (67). miR-27a has two mature forms: miR-27a-5p and miR-27a-3p (68), and recent research indicates that they are overexpressed in gastric cancer, with miR-27a-3p enhancing gastric cancer cell proliferation and tumor growth by modulating BTG2 (69). Currently, there are limited studies on miR-27a-5p regulation, with sparse mentions across various cancers, including breast (70), endometrial (71), gastric (72), lung (73), prostate (74) and ovarian cancer (75). The transcriptome analysis revealed that miR-27a-5p was notably downregulated following 18β-GA intervention compared to the renal cancer group. We examined the impact of miR-27a-5p on renal cancer cell proliferation. Experimental findings demonstrated that miR-27a-5p overexpression markedly enhanced renal cancer cell proliferation, whereas its knockdown significantly suppressed this proliferative capacity. Overexpression of miR-27a-5p, when combined with drug intervention, can counteract its own enhancement of renal cancer cell proliferation. The experimental findings indicate that 18β-GA suppresses renal cancer cell proliferation by downregulating miR-27a-5p.

Autophagy is an intracellular degradation process characterized by the accumulation of autophagosomes, which is crucial for regulating natural cell death during development and responding to metabolic stress (76). Growing evidence suggests that autophagy plays a role in the development and progression of various cancers, including gastrointestinal malignancies (77), head and neck carcinoma (78), breast cancer (79) and prostate cancer (80). Recent research indicates a significant link between renal cell carcinoma and autophagy-associated proteins, underscoring their potential as therapeutic targets or biomarkers for tumor progression monitoring (81). Our study confirmed this report. Network pharmacology predictions revealed that the targets shared by 18β-GA and renal cancer were enriched in PPAR signaling pathway. All isoforms of PPAR have been shown to modulate autophagy across various disease conditions and cellular responses (82). Specifically, PPARα has been demonstrated to promote autophagy induction and autophagosome maturation, as well as suppress inflammatory pathways (83). PPARδ knockout significantly reduced autophagy markers, indicating its role in autophagy (84). Additionally, PPARγ has also been confirmed to activate autophagy and exert effects on cancer cells (85). Our network pharmacology predictions, supported by these studies, suggest that 18β-GA influences renal cancer progression through autophagy regulation. Clinical studies indicate a negative correlation between autophagy levels and both the stage and grade of renal cell carcinoma. Reduced autophagy, indicated by decreased LC3II expression, is linked to an unfavorable prognosis in patients, implying it may facilitate the progression of renal cell carcinoma (86). LC3 is a recognized marker for autophagic cell death, with its dynamic changes directly indicating the autophagy process. Upon activation of the autophagy pathway, LC3 is initially cleaved by proteases to form LC3-I, which then interacts with phosphatidylethanolamine to convert into membrane-localized LC3-II (87). A recent study suggests that enhancing LC3 expression may inhibit renal cell carcinoma proliferation, as LC3 aggregation on autophagosomes signals autophagy initiation (35), and the ratio of LC3-II to LC3-I expression is widely recognized as a key indicator of autophagy induction (87). Accordingly, we performed a WB assay to detect LC3 protein expression. Results showed that miR-27a-5p knockdown upregulated LC3II levels, suggesting autophagy activation; conversely, miR-27a-5p overexpression reduced LC3II and inhibited autophagy. Notably, 18β-GA administration during miR-27a-5p overexpression reversed this autophagy inhibition. The results suggest that 18β-GA may reduce miR-27a-5p levels, leading to increased LC3II expression, which activates autophagy and inhibits renal cancer cell proliferation, aligning with earlier studies.

The research demonstrated that 18β-GA can trigger autophagy via the miR-27a-5p/LC3 pathway and suppress renal cancer cell proliferation. 18β-GA triggered autophagy through suppression of miR-27a-5p and elevation of LC3-II levels, leading to diminished cell viability in 786-O and ACHN cells, ultimately suppressing renal carcinoma cell proliferation. This research solely confirmed the impact of 18β-GA on renal cancer proliferation at the cellular level. Subsequent animal experiments will be conducted to verify the *in vivo* mechanism of 18β-GA in treating renal cancer. BLI bio-membrane interference and thermal migration technologies will verify the interaction between 18β-GA and autophagy proteins. Co-IP will confirm the interaction between LC3II and the predicted downstream protein ATG3. Additionally, ChIP and dual-luciferase reporter assays will investigate the targeting relationship between miR-27a-5p and LC3, elucidating the specific mechanism of 18β-GA’s anti-tumor activity.

## Data Availability

The original contributions presented in the study are included in the article/**Supplementary Material**. Further inquiries can be directed to the corresponding authors.

## Supplementary material

Supplementary Figure 1 WGCNA analysis and intersection gene analysis. **(A)** Network configuration parameters. **(B)** Gene tree map obtained by average linkage hierarchical clustering. **(C)** Topological overlapping heat map of gene network. **(D)** Correlation heat map of character modules. **(E)** Intersection map of 18β-GA and renal cancer target and WGCNA analysis. Blue represents renal cancer genes, green represents differentially expressed genes in the GEO dataset, pink represents WGCNA genes, and purple represents 18-GA genes. **(F)** Cross-target heat map. Red and green represent the tumor group and the normal group respectively. **(G)** The LogFC values of the top 10 up-regulated and down-regulated genes of the cross-target, where orange and blue represent the up-regulated and down-regulated genes, respectively. **(H)** PCoA plots of intersecting target samples. The orange dots represent the normal group and the green dots represent the tumor group.

Supplementary Figure 2 Core target screening and enrichment analysis. **(A)** Topology screening process of PPI network, the larger the circle and the redder the color, the more important the target is in the network. **(B)** The degree value of the core goal. **(C)** Correlation heatmap of the core targets. Red represents positive correlation, blue represents negative correlation, and darker color indicates stronger correlation. **(D)** GO lollipop plot of intersecting targets. Red, blue and green represent BP, CC and MF respectively, the size of the circle represents the number of enriched targets, the bigger the circle, the more targets are enriched. **(E)** KEGG circle diagram of intersecting targets. The outermost left side of the circle represents the intersection target, the outermost right side color represents the pathway, and the color of the innermost right side of the circle represents the P-value of the enriched pathway; the lighter the color, the more significant the enriched pathway.

Supplementary Figure 3 Hub gene expression and prognosis analysis. **(A)** Immunohistochemistry of the Hub gene in normal renal tissues and renal cancer tissues. Brown indicates the expression level of the Hub gene. **(B)** Fluorescence mapping of Hub gene in tumor tissue. Blue represents the nucleus, red represents microtubule tissue, and green represents the Hub gene. **(C)** Hub gene survival curve. The horizontal coordinate represents the survival time.

Supplementary Figure 4 Relationship between Hub genes and immune infiltration. **(A)** Scatter plot of StromalScore, ImmuneScore, ESTIMATEScore correlation of Hub genes. **(B)** Single-cell sequencing map of Hub genes. **(C)** Heatmap of correlation between Hub genes and immune checkpoints. Blue color represents negative correlation and red color represents positive correlation. **(D)** Hub gene correlates with Macrophage, Monocyte, Cancer associated fibroblast, and CD8+ T cell infiltration. Green represents negative correlation, pink represents positive correlation, and larger bubbles and darker colors represent stronger correlation.

Supplementary Figure 5 Association of Hub genes with antitumor drug therapy. **(A)** Hub gene and immunotherapy network diagram. **(B)** The relationship between the expression level of Hub gene and INF-γ、 TGF-β. **(C)** The correlation between Hub gene and immunotherapy.

Supplementary Figure 6 Toxicity model calculation of 18β-GA. **(A)** Summary of toxicity of 18β-GA. **(B)** Prediction of toxic dose and toxicity grade of 18β-GA. **(C)** Diagram of the network between 18β-GA and predicted activity. **(D)** Toxicity radar map of 18β-GA.

Supplementary Figure 7 The clinical relevance of miR-27a to renal cancer. **(A)** Prediction of miR-27a expression in renal cancer. **(B)** Prediction of miR-27a survival prognosis in renal cancer. **(C)** Prediction of miR-27a survival prognosis in pan-cancer. **(D)** Prediction of miR-27a-5p expression in renal cancer. **(E)** Prediction of miR-27a-5p survival prognosis in renal cancer. **(F)** Prediction of the correlation between miR-27a-5p and LC3A, LC3B, LC3C. **(G)** Correlation diagram of miR-27a-5p related targets and autophagy target.

Supplementary Figure 8 **(A)** Check of sequencing error rate distribution. **(B)** sRNA length screening. **(C)** Distribution density map of reads on each chromosome. **(D)** Box plots and density distribution plots of TPM for different samples. **(E)** Heatmap of microRNA expression correlation among all samples.
