# Correction: Study on the mechanism of 18β-glycyrrhetinic acid inhibiting the proliferation of renal cancer cells by inducing autophagy through the miR-27a-5p/LC3 axis

**DOI:** 10.3389/fonc.2026.1845623

**Published:** 2026-05-13

**Authors:** Shumin Jia, Lei Zhang, Yahong Li, Duojie Xu, Yi Yang, Ziying Zhou, Wenjing Liu, Jianan Zhao, Ling Yuan, Yi Nan

**Affiliations:** 1 Key Laboratory of Dryness Syndrome in Chinese Medicine, Ministry of Education, Ningxia Medical University, Yinchuan, China; 2 Traditional Chinese Medicine College, Ningxia Medical University, Yinchuan, China; 3 College of Pharmacy, Ningxia Medical University, Yinchuan, China

**Keywords:** 18β-glycyrrhetinic acid, autophagy, miR-27a-5p/LC3 axis, proliferation, renal carcinoma

There was a mistake in **Figure 14** as published. During a post-publication self-check, we identified that due to an oversight in figure arrangement and typesetting, the image originally belonging to the second row of **Figure 13A** was incorrectly placed as the first image in the second row of **Figure 14A**. The corrected **Figure 14** appears below.

**Figure 14:**
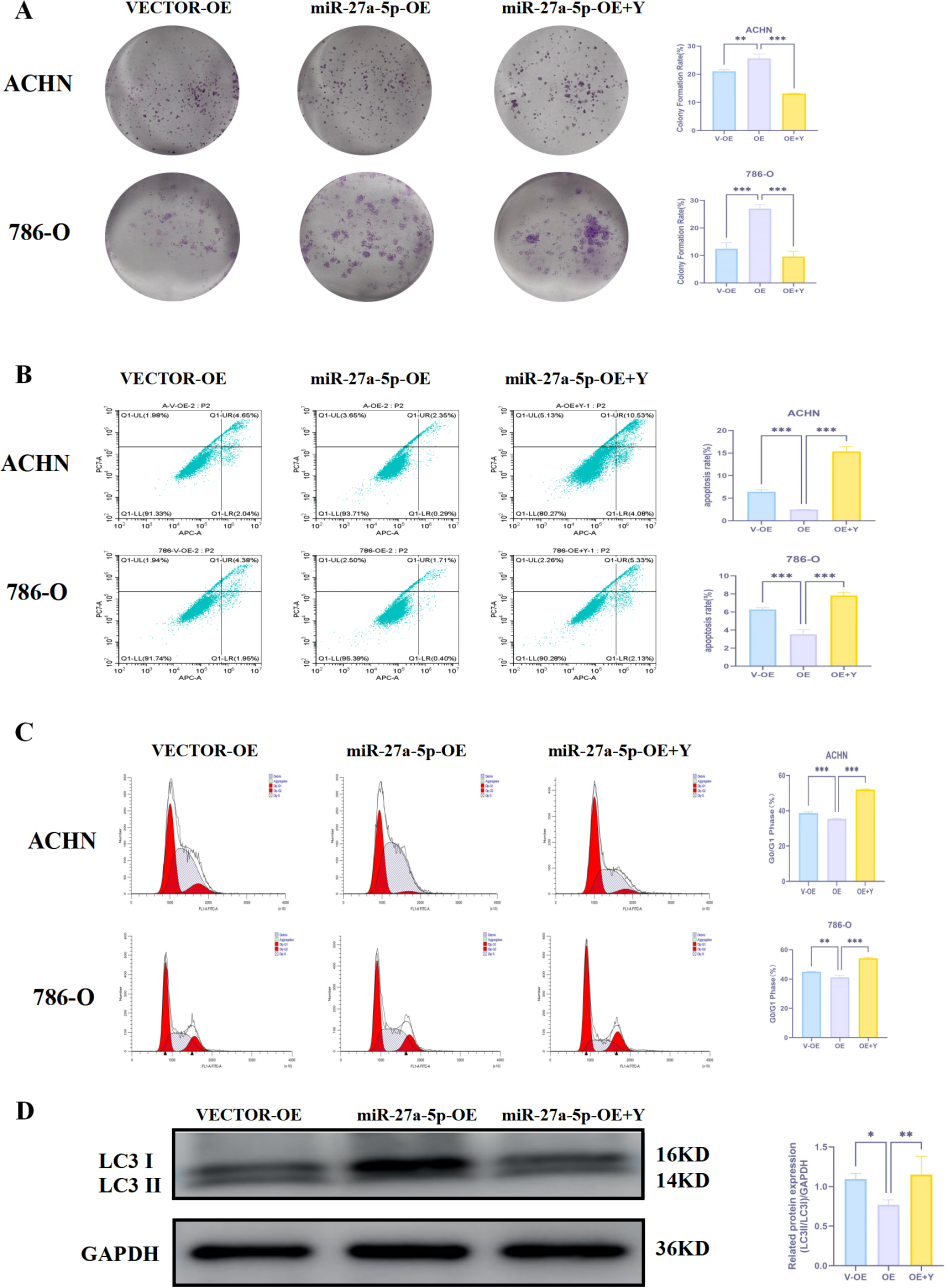
18β-GA regulates the effect of miR-27a-5p on the proliferation phenotype of human renal cancer cells. **(A)** 18β-GA regulates the effect of miR-27a-5p on the clone formation ability of human renal cancer cells. **(B)** 18β-GA regulates the effect of miR-27a-5p on the apoptosis of human renal cancer cells. **(C)** 18β-GA regulates the effect of miR-27a-5p on the cell cycle of human renal cancer cells. **(D)** 18β-GA regulates the effect of miR-27a-5p on the expression of LC3 protein in human renal cancer cells. *p < 0.05, **p < 0.01, ***p < 0.001 indicate that the results are statistically significant.

The original version of this article has been updated.

