# Supplementary figures and images for: Study on the mechanism of 18β-glycyrrhetinic acid inhibiting the proliferation of renal cancer cells by inducing autophagy through the miR-27a-5p/LC3 axis
Source: Front Oncol. 2026 Feb 27;16:1762770. doi: 10.3389/fonc.2026.1762770 (PMC12982104; doi:10.3389/fonc.2026.1762770)

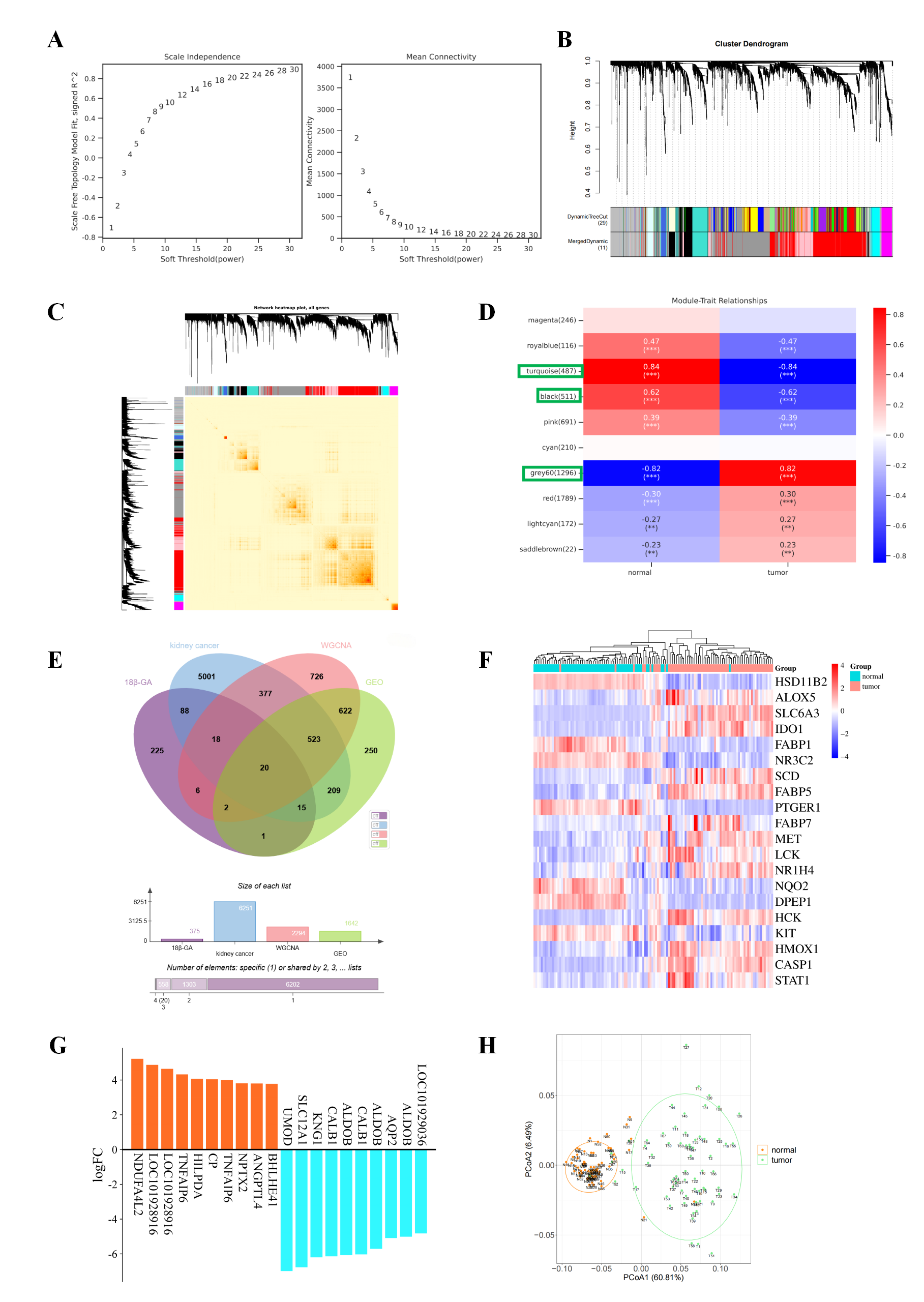

Supplement: Supplementary Figure 1 — WGCNA analysis and intersection gene analysis. (A) Network configuration parameters. (B) Gene tree map obtained by average linkage hierarchical clustering. (C) Topological overlapping heat map of gene network. (D) Correlation heat map of character modules. (E) Intersection map of 18β-GA and renal cancer target and WGCNA analysis. Blue represents renal cancer genes, green represents differentially expressed genes in the GEO dataset, pink represents WGCNA genes, and purple represents 18-GA genes. (F) Cross-target heat map. Red and green represent the tumor group and the normal group respectively. (G) The LogFC values of the top 10 up-regulated and down-regulated genes of the cross-target, where orange and blue represent the up-regulated and down-regulated genes, respectively. (H) PCoA plots of intersecting target samples. The orange dots represent the normal group and the green dots represent the tumor group. [file Image1.tif]

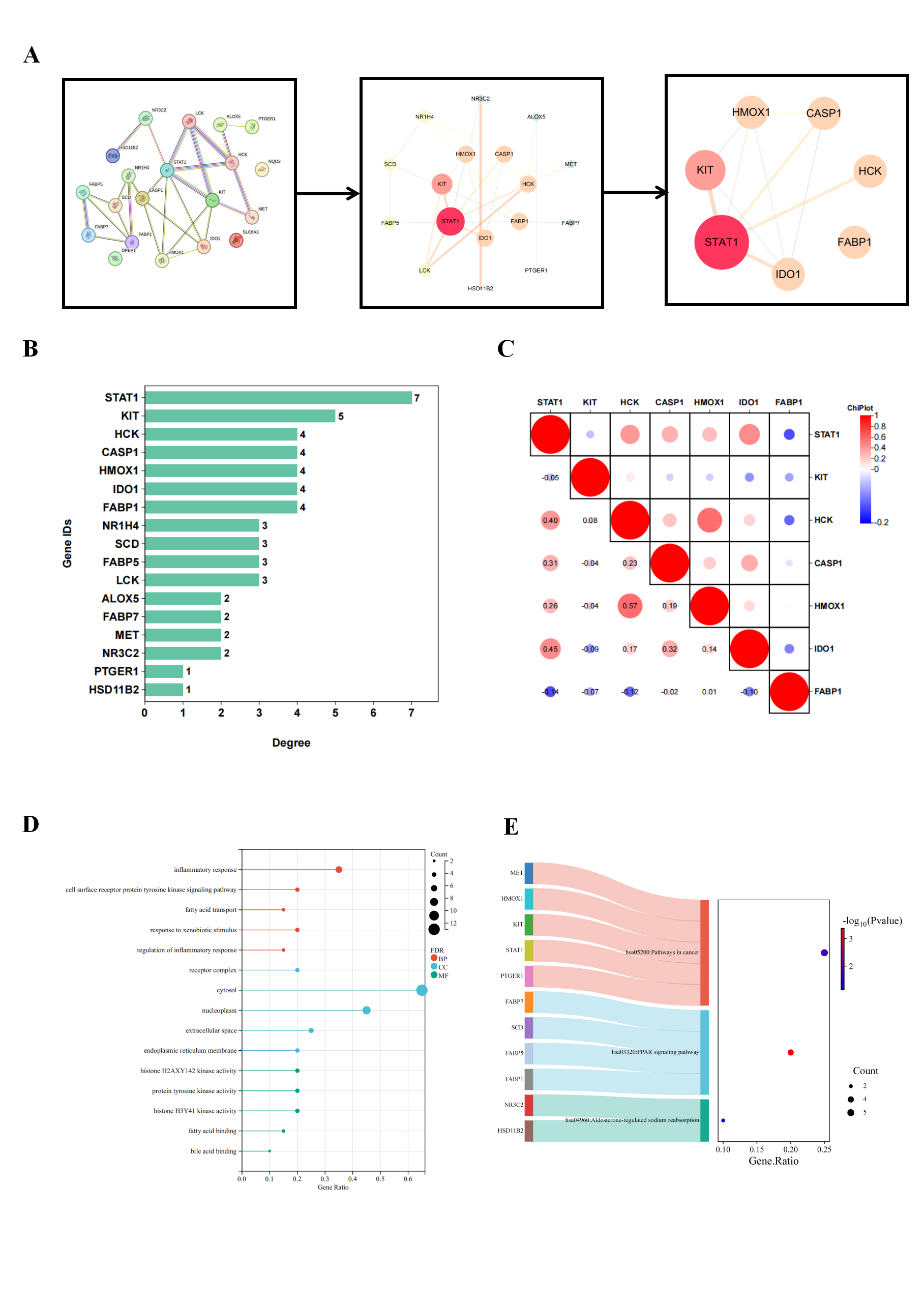

Supplement: Supplementary Figure 2 — Core target screening and enrichment analysis. (A) Topology screening process of PPI network, the larger the circle and the redder the color, the more important the target is in the network. (B) The degree value of the core goal. (C) Correlation heatmap of the core targets. Red represents positive correlation, blue represents negative correlation, and darker color indicates stronger correlation. (D) GO lollipop plot of intersecting targets. Red, blue and green represent BP, CC and MF respectively, the size of the circle represents the number of enriched targets, the bigger the circle, the more targets are enriched. (E) KEGG circle diagram of intersecting targets. The outermost left side of the circle represents the intersection target, the outermost right side color represents the pathway, and the color of the innermost right side of the circle represents the P-value of the enriched pathway; the lighter the color, the more significant the enriched pathway. [file Image2.tif]

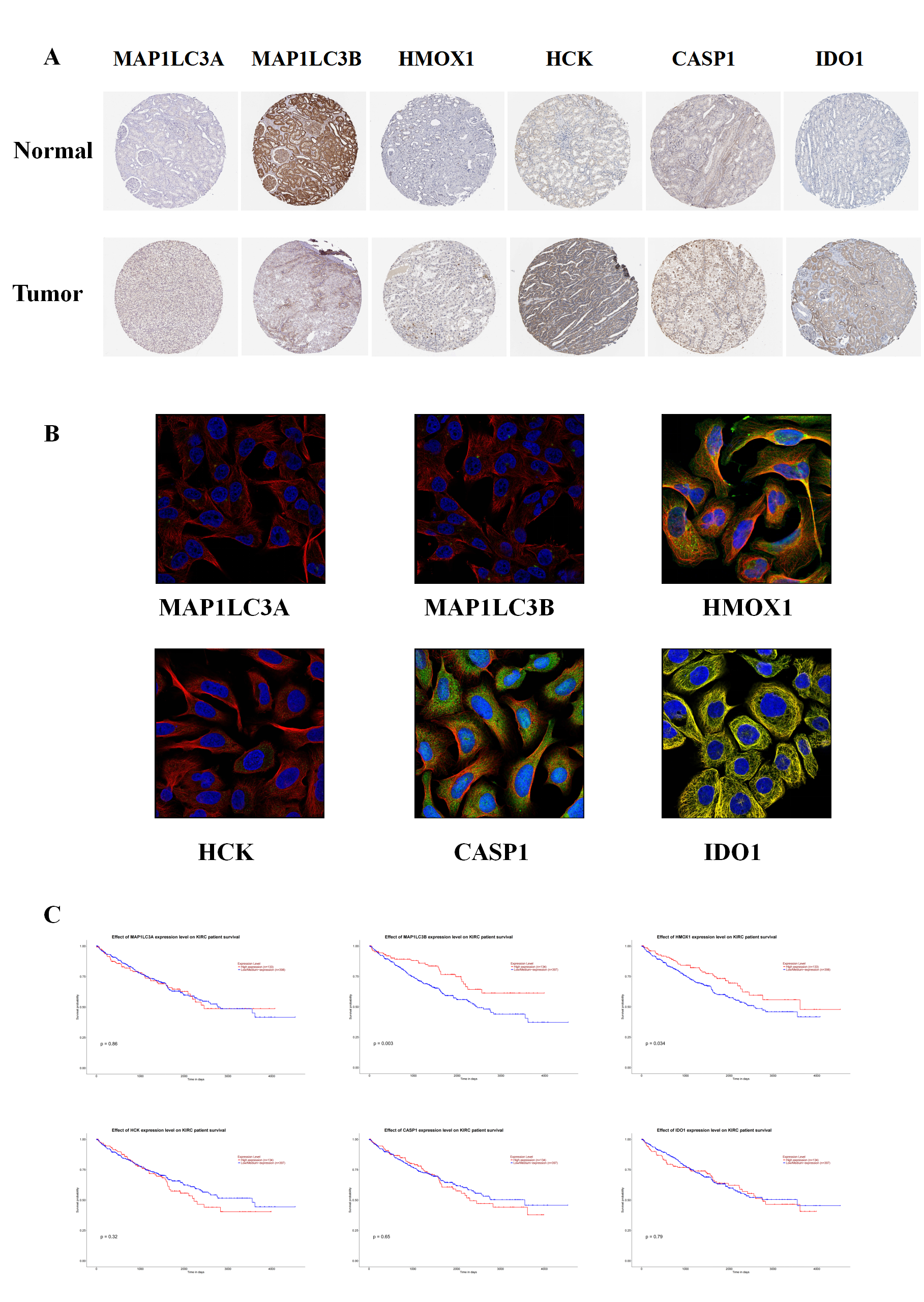

Supplement: Supplementary Figure 3 — Hub gene expression and prognosis analysis. (A) Immunohistochemistry of the Hub gene in normal renal tissues and renal cancer tissues. Brown indicates the expression level of the Hub gene. (B) Fluorescence mapping of Hub gene in tumor tissue. Blue represents the nucleus, red represents microtubule tissue, and green represents the Hub gene. (C) Hub gene survival curve. The horizontal coordinate represents the survival time. [file Image3.tif]

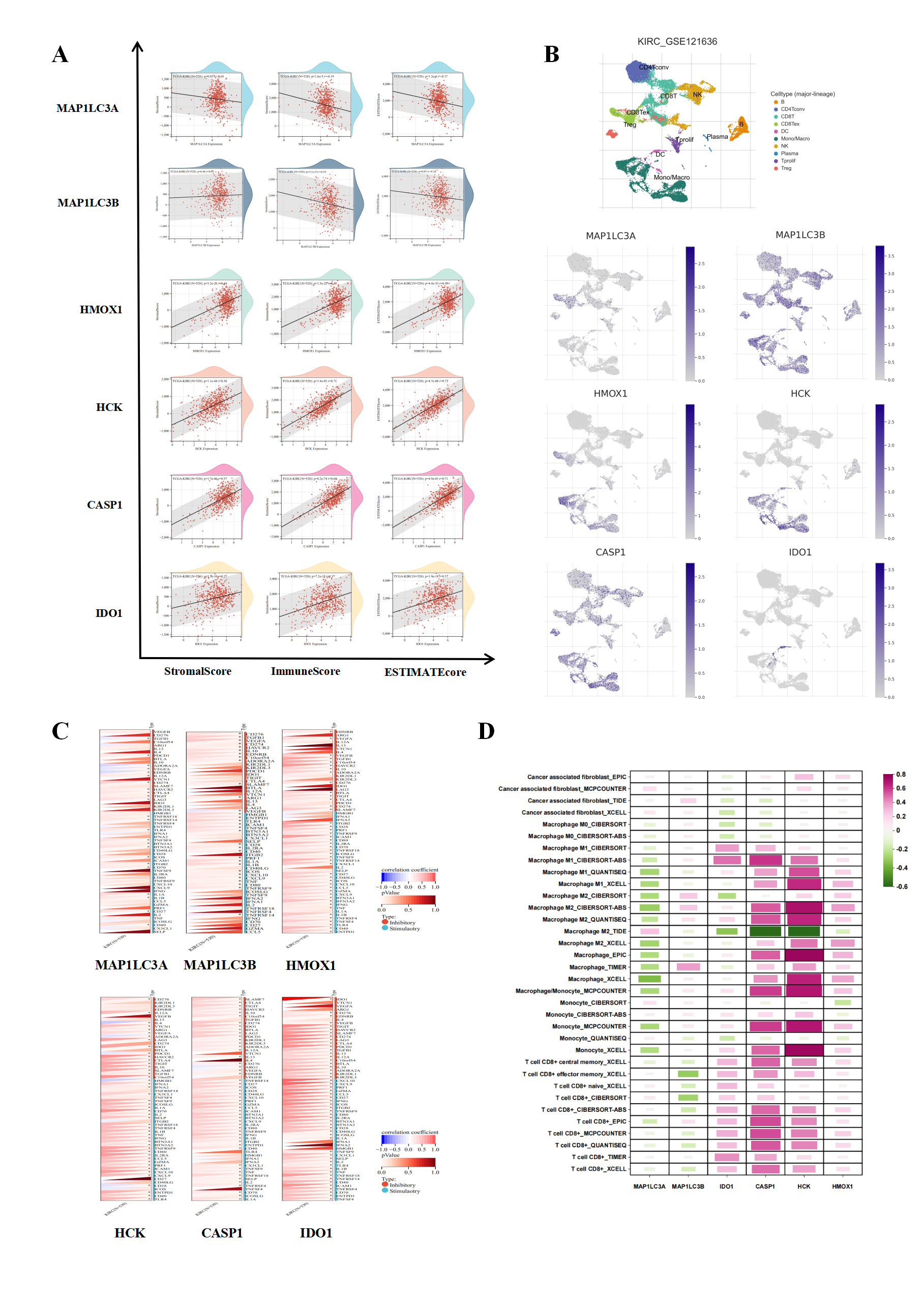

Supplement: Supplementary Figure 4 — Relationship between Hub genes and immune infiltration. (A) Scatter plot of StromalScore, ImmuneScore, ESTIMATEScore correlation of Hub genes. (B) Single-cell sequencing map of Hub genes. (C) Heatmap of correlation between Hub genes and immune checkpoints. Blue color represents negative correlation and red color represents positive correlation. (D) Hub gene correlates with Macrophage, Monocyte, Cancer associated fibroblast, and CD8+ T cell infiltration. Green represents negative correlation, pink represents positive correlation, and larger bubbles and darker colors represent stronger correlation. [file Image4.tif]

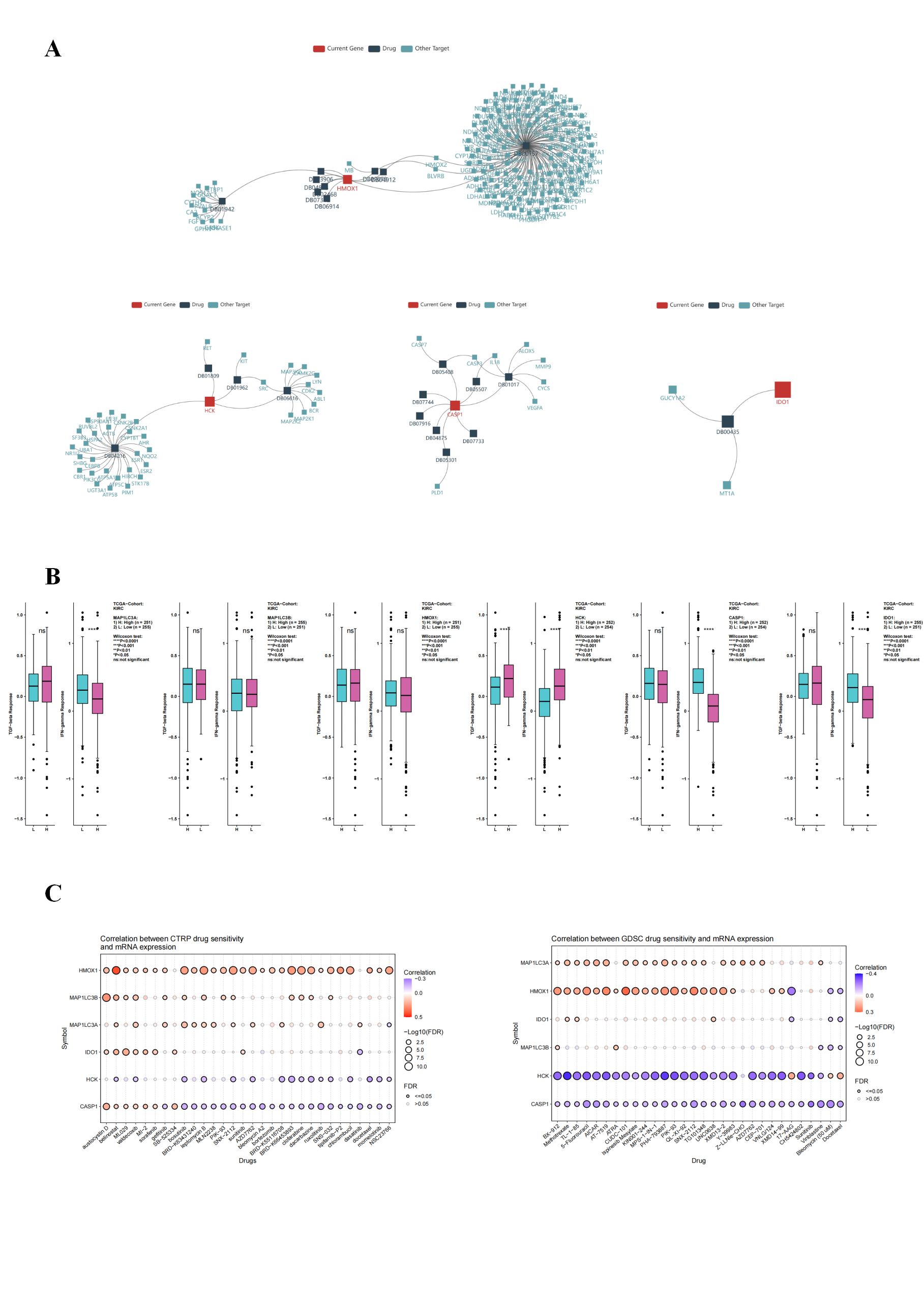

Supplement: Supplementary Figure 5 — Association of Hub genes with antitumor drug therapy. (A) Hub gene and immunotherapy network diagram. (B) The relationship between the expression level of Hub gene and INF-γ、 TGF-β. (C) The correlation between Hub gene and immunotherapy. [file Image5.tif]

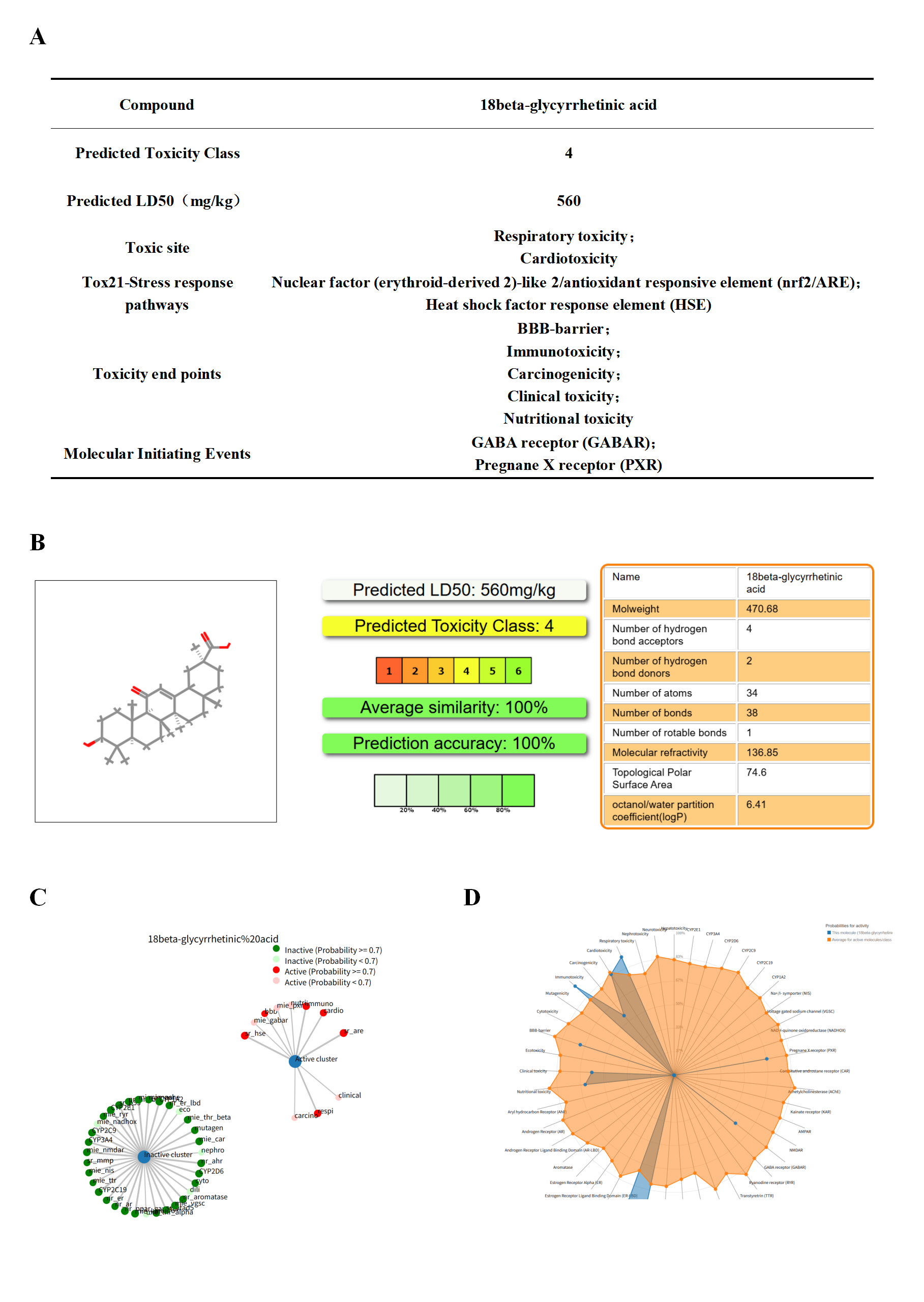

Supplement: Supplementary Figure 6 — Toxicity model calculation of 18β-GA. (A) Summary of toxicity of 18β-GA. (B) Prediction of toxic dose and toxicity grade of 18β-GA. (C) Diagram of the network between 18β-GA and predicted activity. (D) Toxicity radar map of 18β-GA. [file Image6.tif]

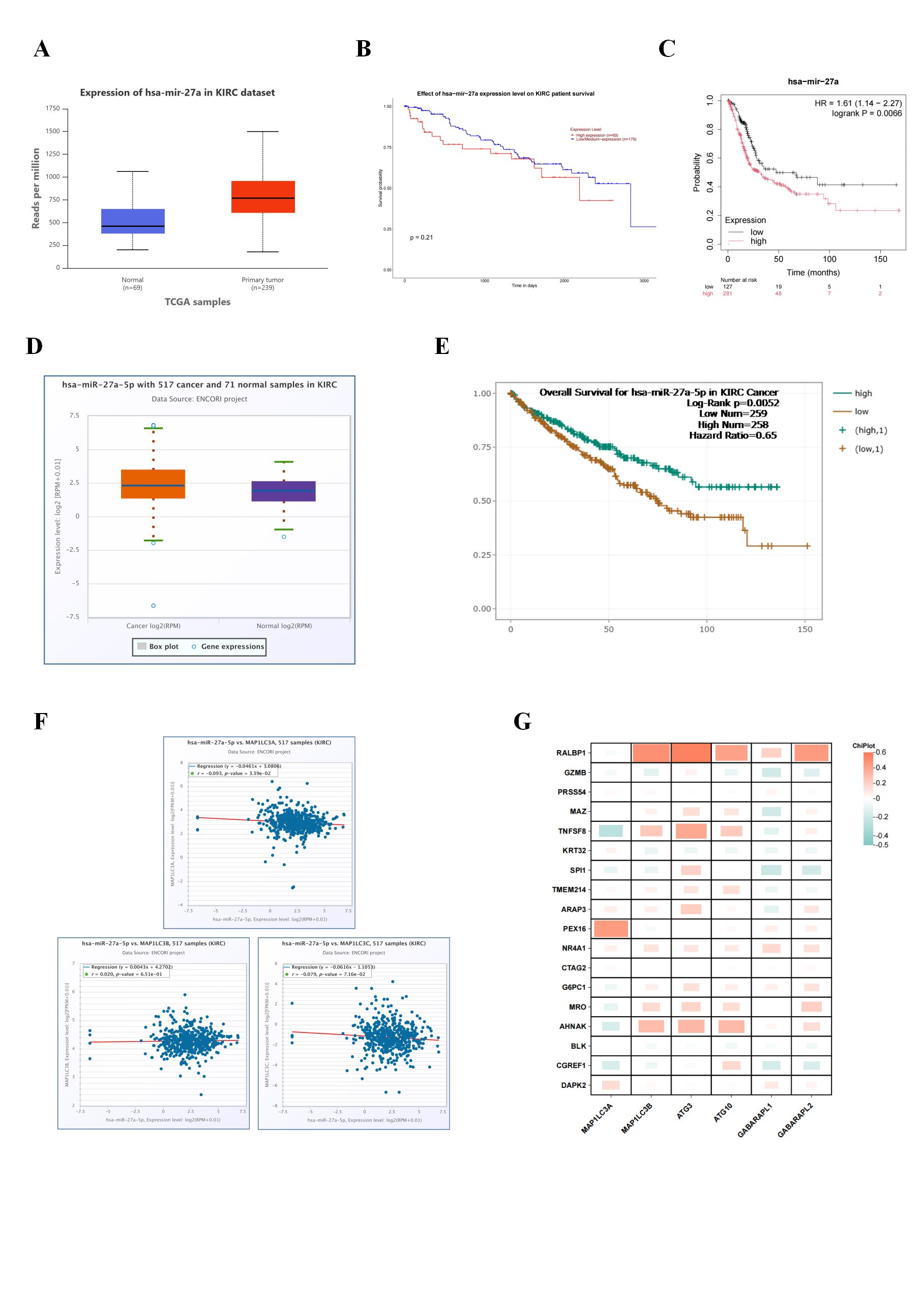

Supplement: Supplementary Figure 7 — The clinical relevance of miR-27a to renal cancer. (A) Prediction of miR-27a expression in renal cancer. (B) Prediction of miR-27a survival prognosis in renal cancer. (C) Prediction of miR-27a survival prognosis in pan-cancer. (D) Prediction of miR-27a-5p expression in renal cancer. (E) Prediction of miR-27a-5p survival prognosis in renal cancer. (F) Prediction of the correlation between miR-27a-5p and LC3A, LC3B, LC3C. (G) Correlation diagram of miR-27a-5p related targets and autophagy target. [file Image7.tif]

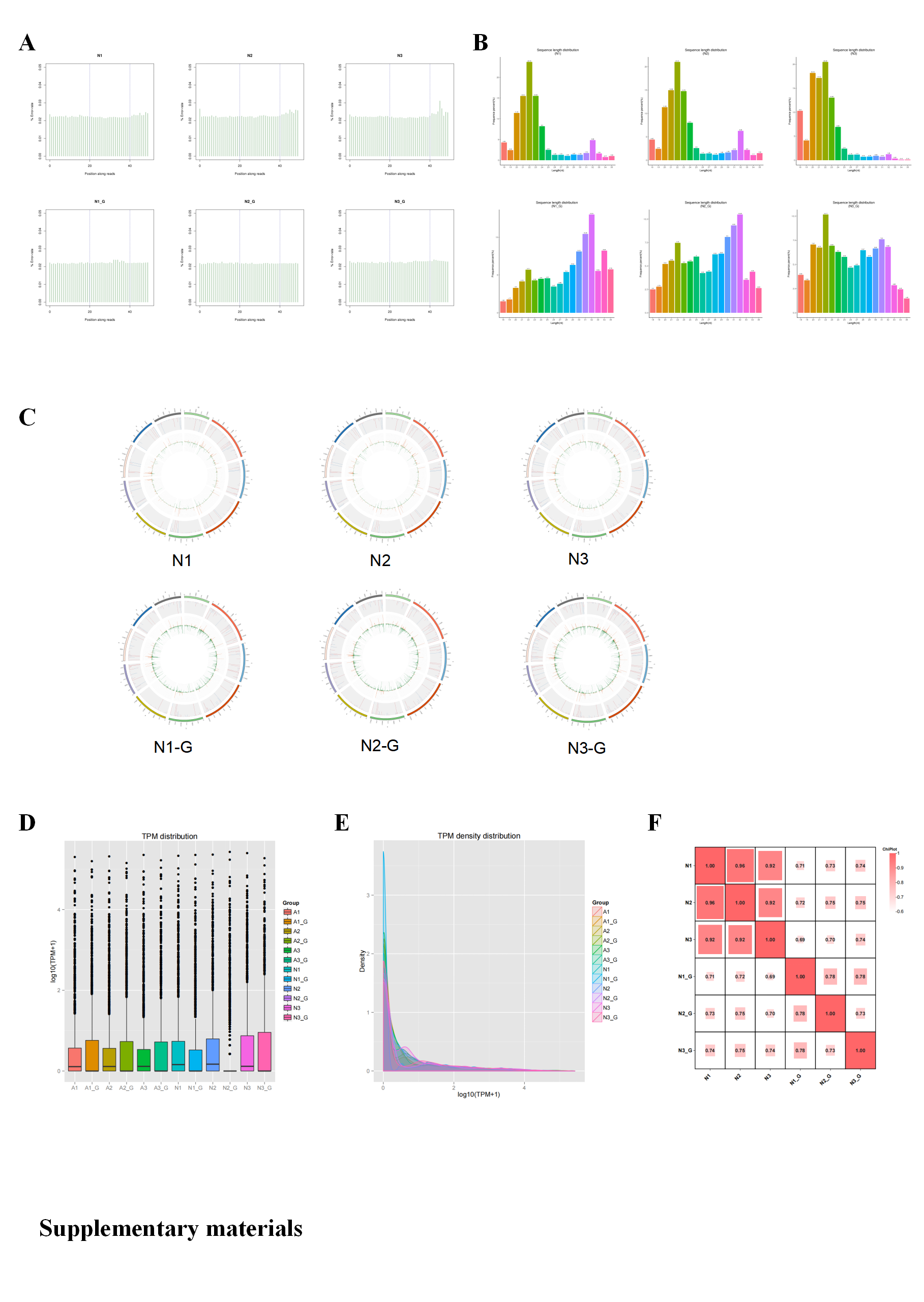

Supplement: Supplementary Figure 8 — (A) Check of sequencing error rate distribution. (B) sRNA length screening. (C) Distribution density map of reads on each chromosome. (D) Box plots and density distribution plots of TPM for different samples. (E) Heatmap of microRNA expression correlation among all samples. [file Image8.tif]
